# Multi‐Omics Analysis in Autoimmunity: Identification of MicroRNA Regulatory Networks and Cell‐Type‐Specific Dysregulations in Multiple Sclerosis and Type 1 Diabetes

**DOI:** 10.1002/mco2.70647

**Published:** 2026-04-02

**Authors:** Jacopo Ronchi, Roberta Rigolio, Davide Maria Trevisan, Angela Papagna, Angela Stabilini, Martina Gallinaro, Maria Letizia Fusco, Martina Gaia Cogo, Guido Cavaletti, Giovanni Malerba, Manuela Battaglia, Maria Foti

**Affiliations:** ^1^ School of Medicine and Surgery University of Milano‐Bicocca Monza MB Italy; ^2^ PhD Program in Neuroscience Medicine and Surgery Department University of Milano‐Bicocca Monza Italy; ^3^ Diabetes Research Institute IRCCS San Raffaele Scientific Institute Milan Italy; ^4^ Department of Neurosciences Biomedicine and Movement Sciences University of Verona Verona Italy; ^5^ Fondazione IRCCS San Gerardo Dei Tintori Monza Italy; ^6^ BicOMICs University of Milano‐Bicocca Monza MB Italy

**Keywords:** autoimmunity, data integration, microRNAs, multi‐omics, multiple sclerosis, Type 1 diabetes

## Abstract

Autoimmune diseases (AIDs) arise from complex immune dysregulations involving multiple immune cell types, cytokines, and molecular mediators. Among these, microRNAs (miRNAs) have recently emerged as key regulators of leukocyte processes and are frequently dysregulated in AIDs. However, their role in disease pathophysiology remains poorly understood. In this study, we performed a comprehensive analysis of miRNA expression in three immune populations, namely, CD14^+^ monocytes, neutrophils, and CD8^+^ T cells, in multiple sclerosis (MS) and type 1 diabetes (T1D), two prototypical AIDs. Our results reveal distinct patterns of miRNA dysregulation in each cell type, with monocytes from T1D patients showing enhanced M1 polarization and supporting inflammatory vascular damage. On the other hand, CD8^+^ T cells from MS patients show profound alterations related to CD8^+^ T cell‐fate commitment, apoptosis regulation, and migratory capacity. Notably, we identified miRNAs that regulate key transcription factors such as FOXP3, IRF4, and RORγt, potentially shaping T cell differentiation programs. Our results suggest that miRNA networks play a central role in orchestrating disease‐specific dysregulation in AIDs. By elucidating these intricate regulatory mechanisms, our study provides a foundation for future therapeutic strategies targeting miRNAs in autoimmunity.

## Graphical abstract

This study evaluates the expression of microRNAs (miRNAs) in three distinct immune subsets (CD14^+^ monocytes, neutrophils, and CD8^+^ T cells) in multiple sclerosis and type 1 diabetes, reporting the presence of unique alteration patterns. Our integrative multi‐omics analysis suggests that miRNAs play a pivotal role in the differentiation and migration of CD8^+^ T cells, the polarization of monocytes, and the induction of vascular damage. These findings underscore the central role of miRNAs in immune dysregulations that underpin autoimmune diseases and reveal potential therapeutic implications.

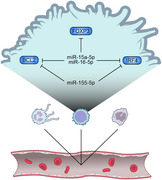

## 1 Introduction

Autoimmune diseases (AIDs) are complex disorders that manifest in approximately 4–5% of the general population. Despite the extensive research conducted in the field, a comprehensive characterization of the dysregulatory events remains elusive, probably because of the substantial heterogeneity observed among AID patients. In this context, recent advancements in multi‐omics research have proven effective in deciphering the intricate molecular networks underlying different human disorders [1, 2], thereby facilitating the derivation of clinically meaningful insights, including patient stratification, biomarker distribution, and therapeutic target identification.

Among AIDs, multiple sclerosis (MS) is a neurological disease of the central nervous system (CNS) that is characterized by the presence of diffuse demyelinating lesions in the white and gray matter of the brain and spinal cord [3, 4]. The complex interplay of various immune cell types is a hallmark of MS. Among the others, the contribution of various subsets of T lymphocytes has been reported, although their contribution is dynamic and multifaceted, ranging from deleterious to protective. At present, three clinical patterns of disease progression are recognized: relapsing–remitting MS (RRMS), primary progressive MS, and secondary progressive MS [5]. Nevertheless, the boundaries between these clinical phenotypes are not well understood, which limits treatment options and patient stratification. Type 1 diabetes (T1D) is another autoimmune disorder that results in the destruction of insulin‐secreting β cells of the pancreas. The predominant theory posits that the development of T1D is attributable to a combination of environmental, immunological, metabolic, and genetic factors [6, 7]. Also for T1D, a coordinated interaction of numerous cell types, coupled with cytokine release, has been implicated in the propagation and perpetuation of the autoimmune destruction of β cells. Furthermore, the clinical heterogeneity of T1D has garnered significant attention, leading to the proposal of T1D endotypes that likely reflect the presence of multiple pathophysiological mechanisms.

MS and T1D share several characteristics, including the presence of circulating autoantibodies and the infiltration of autoreactive lymphocytes and myeloid cells within affected tissue. The fundamental molecular mechanisms underlying both MS and T1D have remained elusive; however, they appear to involve the combination of specific susceptibility genes with environmental influences, resulting in a breakdown of T cell‐dependent and B cell‐dependent self‐tolerance. The complex relationship between genetic predispositions and environmental influences is likely modulated by epigenetic mechanisms, such as DNA methylation, histone modifications, and noncoding RNAs, including microRNAs (miRNAs). In recent years, there has been significant research interest in the field of miRNAs and their role in the immune system, particularly in the context of AIDs. Specifically, aberrant expression of miRNAs has been shown to regulate the differentiation and activation of immune cells in autoimmunity [8]. Nonetheless, elucidating the precise roles of specific miRNAs in diverse pathophysiological contexts remains challenging due to the enigmatic nature of this regulatory machinery.

In this study, we sought to disclose the impact of miRNA dysregulations in MS and T1D by employing an integrated multi‐omics approach. To this end, we used a combination of omics experiments and miRNA profiling to comprehensively investigate the disrupted miRNA–mRNA regulatory networks in three distinct cellular types for each AID. In particular, CD8^+^ T cells were examined for the lymphocyte compartment, while monocytes and neutrophils were assessed for the myeloid counterpart.

The findings of this study will contribute to the identification of the functional consequences of dysregulated miRNAs in these cell types for both MS and T1D. Furthermore, our integrative multi‐omic approach allowed the discovery of the miRNA‐regulatory networks that influence the activity of CD8^+^ T cells in RRMS patients. This strategy will facilitate the investigation of the molecular mechanisms underlying AID pathogenesis and will pave the way for the identification of novel therapeutic approaches.

## 2 Results

### 2.1 MiRNA Dysregulation is Most Evident in CD8^+^ T Cells in MS and CD14^+^ Cells in T1D

Blood samples were collected from a total of 61 subjects, divided into two cohorts. Individuals in the first cohort were recruited at San Gerardo Hospital (HSG) in Monza, comprising 20 RRMS patients and 15 age‐matched controls. The second cohort was enrolled at San Raffaele Hospital (HSR) in Milan, and included 12 patients with T1D and 14 age‐matched controls. Table 1 summarizes the number of subjects used in the analysis along with the main clinical characteristics.

**TABLE 1:** Clinical details of RRMS, T1D, and HC recruited subjects.

	HSG		HSR	
	HC	RR	HC	T1D
Number	15	20	14	12
Sex (M/F)	4/11	6/14	5/9	6/6
Age^[Link]^, ^b^	42.8 (±10.5)	43.6 (±8.6)	31.1 (±6.5)	33.8 (±15.5)
EDSS^a^	—	1.5 (±0.8)	—	—
Duration^[Link]^, ^b^	—	4.9 (±4.4)	—	1.2 (±1.2)

*Abbreviations*: HSG, San Gerardo Hospital; HSR, San Raffaele Hospital; EDSS, expanded disability status scale.

^a^ Both age and duration are expressed in years.

^b^ Data expressed as mean ± SD.

CD14^+^ monocytes, neutrophils, and CD8^+^ T cells were then isolated from each individual donor. After discarding 17 samples due to low RNA quality, a total of 166 miRNA expression profiles were obtained through microarray technology. Moreover, 17 arrays that did not meet the default quality thresholds were excluded from downstream analysis (Table S1).

**FIGURE 1:**
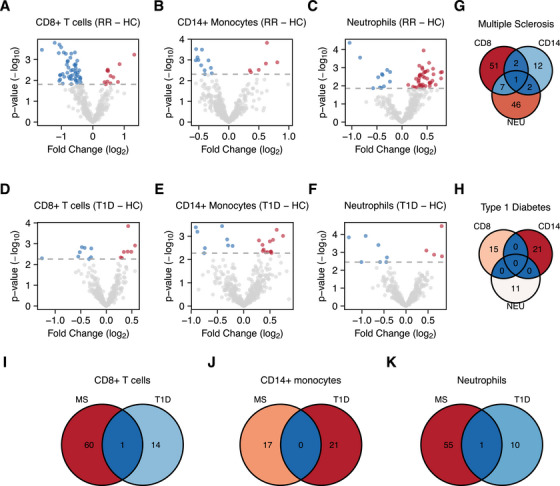
Differentially expressed miRNAs in different cell types from MS and T1D patients. Volcano plots of DE‐miRNAs in CD8^+^ T cells, CD14^+^ monocytes, and neutrophils in relapsing–remitting (RR) MS (A–C) and in T1D (D–F). The horizontal coordinates indicate log_2_ FC, while vertical coordinates indicate ‐log_10_ *p*. Blue dots indicate significantly downregulated miRNAs, whereas red dots represent significantly upregulated miRNAs. Venn diagrams for the common DE‐miRNAs observed for the different cell types in MS and T1D are reported in panels (G) and (H), while common DE‐miRNAs between the two diseases for each cell type are presented in panels (I)–(K).

As illustrated in multidimensional scaling (MDS) plots (Figure S1A–F), it was not possible to define a clear separation between miRNA profiles of MS, T1D, and healthy control (HC) groups. However, after batch‐effect correction, an evident separation based on clinical phenotypes was observed (Figure S2A–F). The results of the differential expression analysis between RRMS and T1D samples compared with healthy subjects are reported in Figure 1. The study identified 61 differentially expressed miRNAs (DE‐miRNAs) in CD8^+^ T cells (*q* < 0.05), 17 in CD14^+^ monocytes (*q* < 0.1), and 56 in neutrophils (*q* < 0.05) for RRMS patients (Figure 1A–C). In contrast, for T1D, a total of 15, 21, and 11 DE‐miRNAs (*q* < 0.1) were defined for CD8^+^ T cells, CD14^+^ monocytes, and neutrophils, respectively (Figure 1D–F). The complete lists of DE‐miRNAs are reported in Tables S2–S7. Collectively, these results indicate dysregulation of miRNAs in both diseases, albeit at different levels.

To ascertain whether the DE‐miRNAs were exclusive to a particular disease or cell type, the intersection between them was considered. The results demonstrated that the majority of dysregulated miRNAs were specific to the cell type within each disease (Figure 1G,H). This finding was further corroborated when the same cell type was compared across the two diseases (Figure 1I–K). However, 12 miRNAs were commonly dysregulated in multiple cell types in MS, while no commonly dysregulated miRNAs were found for T1D (Figure 1G,H).

### 2.2 MiRNA Dysregulations in T1D Regulate CD8^+^ T Cell Activation, Support Inflammatory Responses, and Prevent Monocyte Apoptosis

**FIGURE 2:**
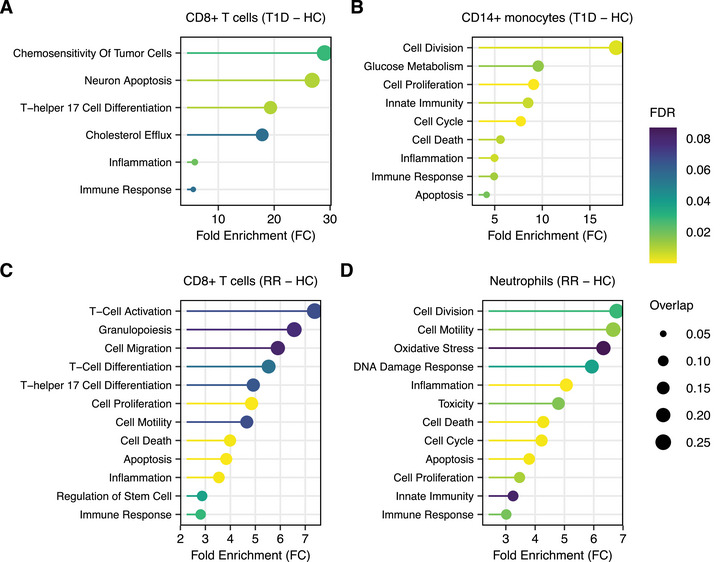
Functional enrichment analysis of DE‐miRNAs in MS and T1D. Panels (A) and (B) show the molecular processes affected by miRNA dysregulation in CD8^+^ T cells and CD14^+^ monocytes from T1D patients, respectively. Conversely, panels (C) and (D) show the pathways that are impaired in CD8^+^ T cells and neutrophils isolated from patients with relapsing–remitting (RR) MS.

To explore the biological roles of miRNA dysregulation, we conducted an overrepresentation analysis using the miRNA sets provided by the TAM 2.0 database. Regarding T1D, we report the enrichment of the “Th17 cell differentiation” category (Figure 2A). Although T helper 17 cells (Th17) are a subset of CD4^+^ T cells, IL‐17‐producing CD8^+^ T (Tc17) cells have recently been described in the context of autoimmunity [9] and are increased in children with new‐onset T1D [10]. In detail, we observed the downregulation of miR‐27a‐3p, miR‐27b‐3p, and miR‐29a‐3p, which have been demonstrated to promote Th17 differentiation [11], coupled with an increase in the expression of miR‐20b‐5p, which has been shown to impede Th17 differentiation [11]. Furthermore, the downregulation of miR‐27a‐3p and miR‐27b‐3p resulted in an enrichment of the “cholesterol efflux” category (Figure 2A). These two miRNAs have been demonstrated to target the ATP Binding Cassette A1 (ABCA1), which is a cholesterol transporter that mediates cholesterol efflux to apoA‐I [12]. Notably, increased cholesterol efflux via ABCA1 has been associated with increased T cell proliferation and diminished apoptosis [13].

Besides CD8^+^ T cells, the functional consequences of miRNA dysregulations in purified monocytes were also investigated (Figure 2B). Among the miRNAs involved in the regulation of the immune response, we observed the upregulation of miR‐193a‐5p, which drives M1 polarization by suppressing the fatty acid desaturase 1 (FADS1) [14, 15]. Furthermore, we observe the downregulation of miR‐142‐5p, which may result from its excessive secretion within monocyte‐derived extracellular vesicles (EVs) [16, 17]. This is especially relevant considering that the presence of this miRNA within EVs is able to mediate vascular damage in diabetic patients [16]. Ultimately, the data indicate an enrichment of miRNAs involved in apoptosis and cell proliferation (Figure 2B). This phenomenon can be attributed, at least in part, to the downregulation of miR‐16‐5p, miR‐142‐5p, and miR‐140‐3p, which results in increased levels of BCL2 expression and cell proliferation [18, 19, 20]. With regard to neutrophils in T1D, no significant categories were identified (Figure S3).

### 2.3 MiRNA Impairment as a New Player in MS Pathophysiology

Regarding MS, one noteworthy immunological process influenced by miRNA dysregulation is, once again, the “T‐helper 17 cell differentiation” pathway (Figure 2C), which plays a pivotal role in MS‐associated inflammation. In detail, functional enrichment analysis suggests that the downregulation of miR‐93‐3p and miR‐152‐3p, which are two Th17 suppressing miRNAs [11], may coordinate IL‐17 production in CD8^+^ T cells. Notably, Tc17 have been documented to be present at elevated frequencies in both the CSF and peripheral blood of patients with RRMS, and the abundance of this subset is positively correlated with disease activity [21]. Interestingly, the “T‐cell differentiation” category also shows a significant enrichment, indicating that miRNAs may play a role in regulating CD8^+^ T cell fate. In particular, the diminished expression of both miR‐15a‐5p and miR‐16‐5p may drive the differentiation of CD8^+^ T cells toward a regulatory phenotype, due to the lack of inhibition on IRF4 [22, 23]. The potential expansion of CD8^+^ regulatory T cells (CD8^+^ Tregs) and Tc17 cells in MS is further supported by the downregulation of miR‐142‐3p, which directly targets transforming growth factor beta receptor 1 (TGFBR1) [24], a crucial mediator of TGF‐β signaling that is essential for differentiation into both Tregs [25] and Tc17 [26, 27]. Beyond cell differentiation, miRNAs involved in cell migration and cell motility are overrepresented (Figure 2C). In this regard, it has been demonstrated that the knockdown of miR‐152‐3p, miR‐146a‐5p, and miR‐128‐3p may promote cell migration across various cell types [28, 29, 30]. Finally, the enrichment of terms related to cell death and apoptosis is also observed (Figure 2C). Among the involved miRNAs, the reduction of miR‐15a‐5p and miR‐16‐5p expression is notoriously associated with diminished cell apoptosis resulting from the absence of BCL2 inhibition [18, 31].

While no significant categories were found to be enriched in CD14^+^ monocytes (Figure S3), marked alterations are evident in neutrophils (Figure 2D). In this case, miRNA dysregulation exerts a significant impact on critical aspects of neutrophil biology, including the inflammatory profile, oxidative stress, and the DNA damage response (Figure 2D). With regard to oxidative stress, the downregulation of miR‐140‐5p, miR‐148b‐3p, miR‐145‐5p, and miR‐206, may enhance reactive oxygen species (ROS) production through the inhibition of antioxidant factors [32, 33, 34, 35, 36, 37]. Additionally, the increase of miR‐23a‐5p and miR‐320a levels may also be indicative of diminished ROS production [38, 39]. However, the overexpression of miR‐320a and miR‐27a‐5p has also been shown to induce ROS production [40, 41]. In addition to ROS production, the enrichment of the “DNA damage response” category is notable because of the elevated levels of circulating neutrophil extracellular traps observed in RRMS patients [42]. Last, this cell type also exhibits an enrichment of terms related to cell proliferation, apoptosis, and cell motility (Figure 2D).

### 2.4 MiRNAs Affect Gene Expression in CD8+ T Cells in MS

**FIGURE 3:**
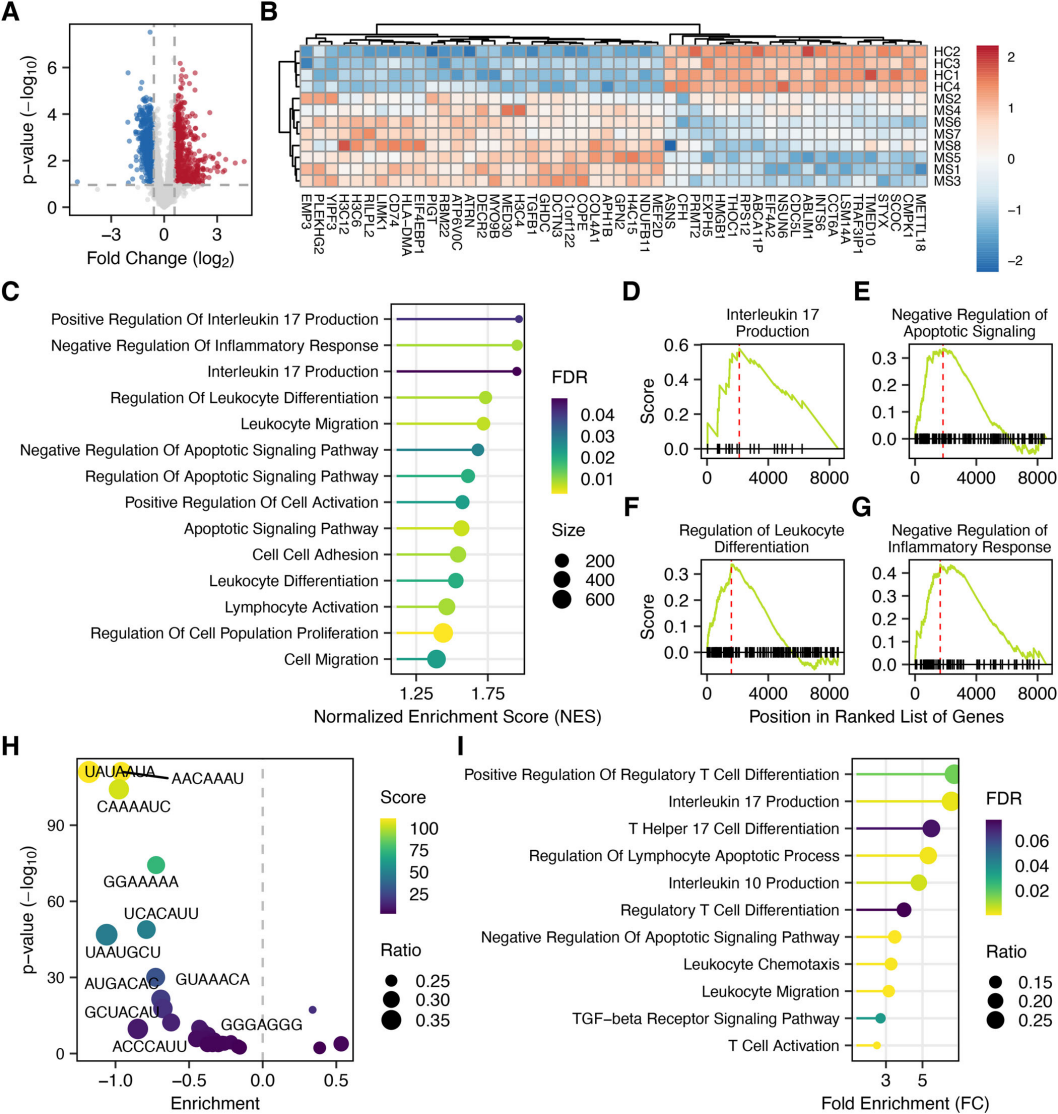
Integrative miRNA–mRNA analysis in CD8^+^ T cells from RRMS patients. Panel (A) displays a volcano plot of DEGs in MS‐derived CD8^+^ T cells. The top 50 genes are shown as a heatmap in panel (B). Panel (C) represents the dysregulated pathways in CD8^+^ T cells according to GSEA. The running GSEA scores for the gene sets involved in IL‐17 production, negative regulation of apoptosis, leukocyte differentiation, and negative regulation of inflammatory response are presented in panels (D)–(G). Panel (H) illustrates the enrichment of miRNA binding sites within DEGs. Each sphere represents a binding motif, and the *x*‐axis indicates its enrichment compared with the frequency expected by chance. Panel (I) shows the GO biological processes affected by influential miRNAs in CD8^+^ T cells based on the overrepresentation of their target genes.

In light of the pivotal function of DE‐miRNAs in CD8^+^ T cells from MS patients, we decided to focus the multi‐omics analysis on this cell type. To this end, a public microarray experiment comparing the CD8^+^ T cell transcriptome of MS patients to that of HCs has been retrieved from the Gene Expression Omnibus (GEO) database (GSE32988). After normalizing probe intensities, lowly expressed genes were removed (Figure S4A), and expression variability was evaluated through MDS plots. The results showed that gene expression values of HCs were well separated from those of RRMS patients (Figure S4B). Following differential expression analysis, 1549 DEGs were identified (*q* < 0.1 and absolute fold‐change > 1.5), of which 657 upregulated and 892 downregulated (Figure 3A and Table S8). The 50 most significant DEGs are displayed in Figure 3B. To infer the affected functions in CD8^+^ T cells, gene‐set enrichment analysis (GSEA) [43] was performed with categories defined in the gene ontology (GO) biological processes database [44].

Notably, we obtained similar findings to those resulting from the functional enrichment of miRNAs, suggesting that miRNAs may play a role in orchestrating the impairment observed at the transcriptomic level. Of particular note is the upregulation of genes implicated in IL‐17 production (Figure 3C,D). As further evidence, TGFB1, one of the most important cytokines for differentiation toward Tc17 [9], is among the overexpressed genes in the MS group (Figure 3B). Moreover, the enrichment of terms related to increased cell proliferation is also evident (Figure 3C). Concurrently, the upregulation of genes that negatively regulate the apoptotic process is observed (Figure 3E). In addition, within the genes involved in leukocyte differentiation (Figure 3F), we observe the overexpression of Treg‐associated genes, including TGFB1, KLF10, CD74, LGALS1, BCL6, IRF4, TOX, and PRDM1, thereby pointing to a potential expansion of CD8^+^ Tregs. This hypothesis is further substantiated by the augmented expression of genes implicated in the negative regulation of the inflammatory process (Figure 3G). Furthermore, we also observe the dysregulation of genes involved in cell migration and T cell activation (Figure 3C).

In order to further investigate the role of miRNAs in these processes, we employed miRNA binding site enrichment analysis to identify binding motifs that are statistically overrepresented in DEGs compared with non‐DEGs. As a result, 28 statistically enriched miRNA binding motifs belonging to the identified DE‐miRNAs were found (adjusted *p* < 0.05). Specifically, the enrichment of upregulated genes reports the overrepresentation of 25 binding sites belonging to 26 downregulated miRNAs (Figure 3H). Conversely, the enrichment of downregulated genes reports the overrepresentation of three binding motifs belonging to three upregulated miRNAs (Figure 3H). The overrepresented binding sites belonging to DE‐miRNAs are reported in Table 2.

**TABLE 2:** DE‐miRNAs that significantly affect gene expression of CD8^+^ T cells from RRMS patients.

Binding site	miRNA	Estimated activity	Size	Overlap	Site overlap	Enrichment^a^	*p* Value	Adjusted *p* value^b^
AACAAAU	hsa‐miR‐7‐1‐3p	Downregulated	2840	981	1990	0.96	8.23 × 10^−112^	1.61 × 10^−109^
UAUAAUA	hsa‐miR‐374b‐5p	Downregulated	2062	812	1400	1.18	1.01 × 10^−111^	1.61 × 10^−109^
CAAAAUC	hsa‐miR‐8063	Downregulated	2670	955	1810	0.98	6.71 × 10^−105^	5.33 × 10^−103^
GGAAAAA	hsa‐miR‐3148	Downregulated	3292	1045	2190	0.72	5.07 × 10^−75^	2.69 × 10^−73^
UCACAUU	hsa‐miR‐23a‐3p	Downregulated	2254	740	1220	0.79	1.36 × 10^−49^	3.59 × 10^−48^
UAAUGCU	hsa‐miR‐155‐5p	Downregulated	1352	522	699	1.06	1.41 × 10^−47^	3.44 × 10^−46^
GUAAACA	hsa‐miR‐30e‐5p	Downregulated	1827	609	852	0.73	8.88 × 10^−31^	1.18 × 10^−29^
AUGACAC	hsa‐miR‐425‐5p	Downregulated	1507	506	654	0.69	3.68 × 10^−22^	3.66 × 10^−21^
GCUACAU	hsa‐miR‐221‐3p	Downregulated	1333	456	557	0.68	1.71 × 10^−18^	1.33 × 10^−17^
ACCCAUU	hsa‐miR‐660‐5p	Downregulated	1123	355	436	0.62	5.66 × 10^−13^	3.67 × 10^−12^
GGAAUGU	hsa‐miR‐206	Downregulated	1787	527	697	0.43	8.54 × 10^−11^	4.93 × 10^−10^
UAGUCCG	hsa‐miR‐6723‐5p	Downregulated	493	178	195	0.85	1.98 ⋅ 10^−10^	1.11 × 10^−9^
GAGAACU	hsa‐miR‐146a‐5p	Downregulated	1642	488	641	0.41	1.76 ⋅ 10^−9^	9.35 × 10^−9^
UUGGCAC	hsa‐miR‐1271‐5p	Downregulated	1665	490	632	0.37	5.39 × 10^−8^	2.72 × 10^−7^
UAGGUGA	hsa‐miR‐6800‐5p	Downregulated	981	297	347	0.45	1.44 × 10^−6^	6.76 × 10^−6^
AGUGGUU	hsa‐miR‐140‐5p	Downregulated	1233	369	442	0.39	2.40 × 10^−6^	1.07 × 10^−5^
GGGUUUA	hsa‐miR‐629‐5p	Downregulated	1470	426	530	0.33	7.27 × 10^−6^	3.17 × 10^−5^
UUCUCCC	hsa‐miR‐629‐3p	Downregulated	2426	654	1010	0.22	3.77 × 10^−5^	1.58 × 10^−4^
AGACUGG	hsa‐miR‐4463	Downregulated	1592	441	571	0.27	8.81 × 10^−5^	3.59 × 10^−4^
CAGUGCA	hsa‐miR‐152‐3p	Downregulated	1740	476	619	0.26	9.75 × 10^−5^	3.93 × 10^−4^
AGUGGAU	hsa‐miR‐6849‐5p	Downregulated	981	284	339	0.34	1.96 × 10^−4^	7.68 × 10^−4^
UGAGUGU	hsa‐miR‐6790‐5p	Downregulated	1229	354	421	0.30	2.15 × 10^−4^	8.32 × 10^−4^
GCCUACU	hsa‐miR‐24‐2‐5p	Downregulated	839	240	279	0.38	2.20 × 10^−4^	8.41 × 10^−4^
CACAGUG	hsa‐miR‐128‐3p	Downregulated	2398	643	912	0.17	1.57 × 10^−3^	5.68 × 10^−3^
AGCAGCA	hsa‐miR‐15a‐5p	Downregulated	2267	602	848	0.15	4.38 × 10^−3^	1.52 × 10^−2^
AGCAGCA	hsa‐miR‐16‐5p	Downregulated	2267	602	848	0.15	4.38 × 10^−3^	1.52 × 10^−2^
GGGAGGG	hsa‐miR‐4728‐5p	Upregulated	3345	789	1880	0.34	5.95 × 10^−18^	2.37 × 10^−16^
CGAGGAG	hsa‐miR‐151a‐5p	Upregulated	483	136	148	0.53	1.44 × 10^−4^	6.75 × 10^−4^
GGGACGG	hsa‐miR‐92b‐5p	Upregulated	447	114	134	0.39	5.80 × 10^−3^	1.85 × 10^−2^

^a^ The odds ratio (OR) for the binding site enrichments is presented in the log_2_ scale.

^b^ *p* Values adjusted for multiple testing through the Benjamini–Hochberg approach.

Collectively, out of the 61 DE‐miRNAs identified in our cohort, 29 of them have a profound effect on target expression, as evidenced by enrichment of their binding sites. Later on, we collected validated miRNA–target interactions from miRTarBase [45] and predicted interactions from the mirDIP database [46]. Overall, we obtained a total of 506,427 interactions between 400 miRNAs and 8570 genes. Subsequently, an overrepresentation analysis was conducted for the identified miRNAs affecting gene expression using the categories provided by the GO database. The results demonstrate that miRNA dysregulations could have a role in almost all the biological processes that are impaired in CD8^+^ T cells (Figure 3I). In particular, we observe the upregulation of target genes, such as IRF4 and SMAD7, which are involved in IL‐17 production and Tc17 differentiation. Furthermore, the increased expression of miRNA targets that are crucial for the differentiation of CD8^+^ Tregs, such as IL2RB and BCL6, is observed. Last, the enhanced expression of genes associated with motility and invasiveness, including CDC42, TLN1, PIP5K1C, CFL1, CORO1C, and MMP9, is indicative of enhanced T‐cell migration. The miRNA–target pairs involving influential miRNAs and their regulated targets participating in Treg differentiation, IL‐10 production, leukocyte chemotaxis, Tc17 differentiation, IL‐17 production, as well as the negative regulation of apoptotic pathways, are shown in Figure S5A–D.

### 2.5 The Downregulation of miR‐15a‐5p and miR‐155‐5p is Responsible for the Overexpression of BCL2 and FOXP3

**FIGURE 4:**
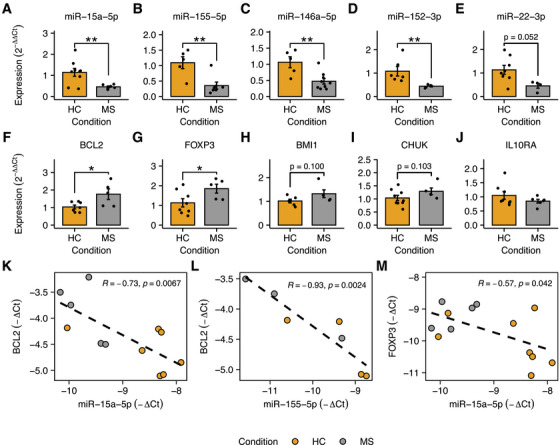
RT‐qPCR validation of DE‐miRNAs and their target genes. Panels (A)–(E) illustrate the relative expression of miR‐15a‐5p, miR‐155‐5p, miR‐146a‐5p, miR‐152‐3p, and miR‐22‐3p in MS patients and controls. Panels (F)–(J) show the relative expression of BCL2, FOXP3, BMI1, CHUK, and IL10RA. Panels (K) and (L) show the negative correlation between ΔCt values of BCL2 and those of miR‐15a‐5p and miR‐155‐5p, respectively. Similarly, panel (M) demonstrates the negative correlation between the expression of miR‐15a‐5p and FOXP3. Ct (cycle threshold) values were obtained by qPCR; relative expression changes are reported as −ΔCt and 2^‐ΔΔCt^ values. *p < 0.05; **p < 0.01.

Given their extensive role in the aforementioned networks, we selected miR‐15a‐5p, miR‐155‐5p, miR‐146a‐5p, miR‐152‐3p, and miR‐22‐3p for further validation. We used microarray expression data to select a housekeeping miRNA that was invariable between conditions, stable across samples, and highly expressed in CD8^+^ T cells. As a result, we identified and selected miR‐150‐5p (CV = 0.06) as the optimal normalizing gene for this analysis. In the comparison between control subjects and MS patients, a statistically significant downregulation was observed for miR‐15a‐5p (*p* = 0.0037), miR‐155‐5p (*p* = 0.0043), miR‐146a‐5p (*p* = 0.0055), and miR‐152‐3p (*p* = 0.003), as shown in Figure 4A–D. Additionally, a near significant downregulation was observed for miR‐22‐3p (*p* = 0.052; Figure 4E).

To confirm the implications of miR‐15a‐5p and miR‐155‐5p downregulation on apoptosis, we tested the expression of BCL2, which is a validated target of both miRNAs. To determine the most suitable housekeeping gene, we evaluated the expression of ACTB, GAPDH, and B2M. ACTB was identified as the most stable gene across all samples (CV = 0.036) and was selected as the normalizer. As displayed in Figure 4F, BCL2 expression was found to be significantly increased in MS patients compared with HCs (*p* = 0.048). Moreover, we proceeded to evaluate the expression of FOXP3, which is also a target of miR‐15a‐5p [47]. Also in this case, a significant increase in FOXP3 expression was observed (*p* = 0.028), as illustrated in Figure 4G. In addition, we evaluated the expression of BMI1 and CHUK, which are two Treg‐related genes that are regulated by miR‐15a‐5p [48, 49, 50]. Although not statistically significant, CD8^+^ T cells from MS patients exhibited an increasing trend in the expression of both BMI1 (*p* = 0.100; Figure 4H) and CHUK (*p* = 0.103; Figure 4I). IL10RA is yet another receptor that plays a role in the functionality of human Tregs [51]. However, we were unable to detect any difference in the expression of this gene (Figure 4J), suggesting a lack of IL10RA modulation in bulk CD8^+^ T cells, at least at the mRNA level. Future experiments are underway to ascertain the modulation at the protein level. Furthermore, as demonstrated in Figure 4K,L, a significant correlation was identified between the expression of BCL2 and that of both miR‐15a‐5p (*R* = −0.73; *p* = 0.0067) and miR‐155‐5p (*R* = −0.93; *p* = 0.0024). Similarly, miR‐15a‐5p significantly correlates with FOXP3 expression (*R* = −0.57; *p* = 0.042; Figure 4M), thus providing evidence for the role of miR‐15a‐5p in fostering the development of CD8^+^ Tregs.

### 2.6 Tc17 Cells and CD8^+^ Treg Are Expanded in Peripheral Blood of RRMS Patients

**FIGURE 5:**
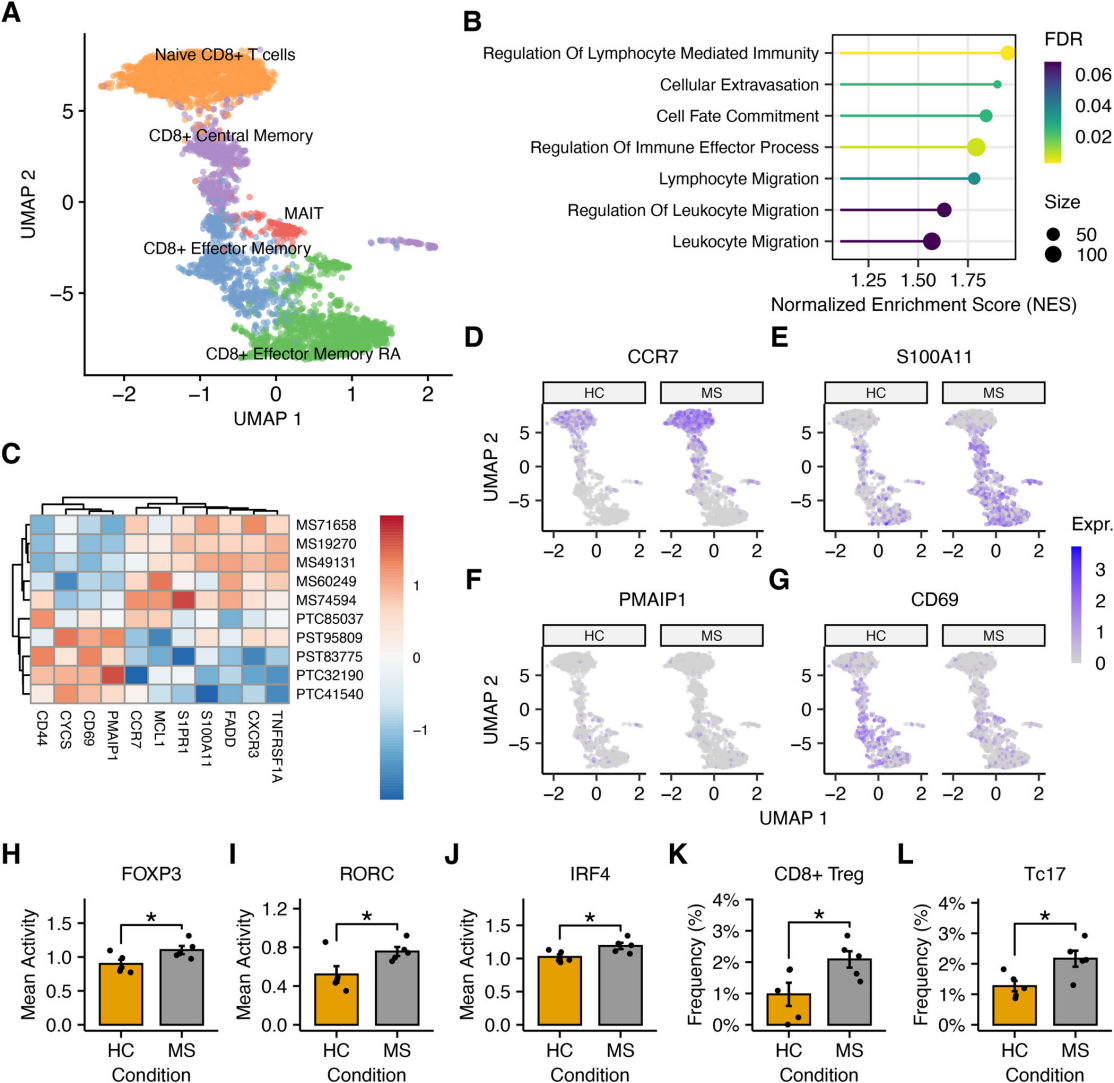
Single‐cell evaluation of CD8^+^ T cells in MS. Panel (A) shows the location of CD8^+^ single‐cell transcriptomes in the UMAP space. Cell clustering and annotation allowed identification of all major subsets of CD8^+^ lymphocytes. Panel (B) shows the functional enrichment analysis of CD8^+^ pseudobulks between RRMS patients and HCs according to GSEA. The differential expression of selected genes is shown in panel (C). Panels (D)–(G) show the differential expression of CCR7, S100A11, PMAIP1, and CD69 in the UMAP space. Expr. indicates gene expression level. Panels (H)–(J) display the mean estimated transcriptional activity of FOXP3, RORC, and IRF4 in patients and controls. Finally, panels (K) and (L) present the expanded frequency of CD8^+^ Tregs and Tc17 cells in the peripheral blood of RRMS patients and HCs, respectively.

To verify our hypotheses, we decided to explore CD8^+^ T cell dysregulations at the single‐cell level. In particular, scRNA‐Seq data of peripheral blood mononuclear cells (PBMCs) from RRMS patients and HCs were obtained from GEO (GSE138266). After isolating CD8^+^ T cells from total PBMCs, the data underwent quality control, normalization, and clustering. The identity of each cluster was automatically defined using SingleR [52] (Figure S6A) and manually refined using the marker expression of each cluster (Figure S6B). The final dataset comprised 4516 CD8^+^ T cells, and the major CD8^+^ T cell subsets were identified (Figure 5A). After computing pseudobulks, MDS plots revealed a clear distinction between RRMS patients and controls (Figure S7A). Subsequent to conducting a differential expression analysis, 393 DEGs were identified, meeting a Storey's *q*‐value threshold of 0.1 (Table S9 and Figure S7B). Consistent with our observations in bulk settings, GSEA revealed the overexpression of genes involved in immune and migratory functions (Figure 5B). This is further substantiated by the upregulation of S1PR1, CXCR3, CCR7, and S100A1 (Figure 5C–E), which are involved in T cell trafficking from and to peripheral tissues [53, 54, 55, 56]. In addition, cells derived from RRMS patients exhibited increased expression of the antiapoptotic modulator MCL1, reduced expression of cytochrome *C*, and reduced expression of NOXA (PMAIP1; Figure 5C,F), which is a proapoptotic member of the BCL2 family. However, increased transcription was observed for both FADD and TNFRSF1A, which may be attributable to the diverse cell‐fates represented within the single‐cell data.

Moreover, CD8^+^ T cells from RRMS patients overexpress genes that are involved in the regulation of lymphocyte activity (Figure 5B), as reflected by the reduced expression of CD44 and CD69 (Figure 5G). Given the evidence for increased regulatory functions, we tried to compare the expression levels of FOXP3. However, due to the low‐depth nature of single‐cell experiments, which resulted in the detection of only a limited number of reads mapping to FOXP3, an alternative strategy was employed. In particular, we estimated the activity of FOXP3 in each cell based on the expression of its targets using multivariate decision trees [57]. After comparing the mean FOXP3 activity, a significant increase in CD8^+^ T cells from RRMS patients was observed (*p* = 0.045; Figure 5H). Although the activity estimation represents an indirect inference rather than a direct measurement, the increased transcriptional activity of FOXP3 is consistent with, and supported by, its elevated expression detected by RT‐qPCR in ex vivo CD8^+^ T cells from our RRMS cohort (Figure 4G). Moreover, as evidenced in Figure 5I, the activity of RORC was found to be significantly higher in RRMS patients (*p* = 0.049), which is highly indicative of increased Tc17 cell differentiation [58]. This is also corroborated by the overactivation of IRF4 (*p* = 0.027; Figure 5J). Likewise, an augmented activity has also been discerned for both STAT3 and RUNX1 (*p* = 0.0039; *p* = 0.0085; Figure S7C,D).

Finally, we quantified the frequency of these rare cell types in the blood of both patients and controls. Nevertheless, due to the lack of consistent expression of marker genes in single‐cell data, reliable identification was not feasible. To address this challenge, we employed AUCell [59], a method that quantifies a score for each cell based on the expression of literature curated signature genes. High scoring cells for each signature were then labeled as either Tc17 cells or CD8^+^ Tregs. To ensure the accuracy of cell identification, we identified marker genes for both these populations and performed an overrepresentation analysis to verify the enrichment of IL‐17 and immune regulatory functions, respectively (Benjamini–Hochberg adjusted *p* < 0.1; Figure S7E,F). After the comparison, the frequency of both CD8^+^ Tregs and Tc17 cells in RRMS patients resulted nearly doubled compared with healthy individuals (*p* = 0.043 for CD8^+^ Tregs and *p* = 0.025 for Tc17 cells), as shown in Figure 5K,L.

## 3 Discussion

Comprehending the intricacies of AIDs poses a considerable challenge, as the immune response is orchestrated by a multifaceted network comprising numerous entities, including immune cells, cytokines, immunoglobulins, and metabolites. Moreover, the immune response to a specific stimulus or infection exhibits significant interindividual variability, resulting in substantial population heterogeneity. Among the molecular species involved, miRNAs have been recently described as central regulators of multiple leukocyte responses, as they have been found dysregulated in different AIDs. Furthermore, they represent attractive targets as a single miRNA can modulate several genes simultaneously. However, before exploiting these small noncoding RNAs for therapeutic interventions, it is imperative to comprehensively understand the molecular events through which they may participate in the development of AIDs. Despite their potential, current understanding of their involvement in the pathophysiology of AIDs remains limited. To address this gap, we have undertaken a holistic approach by simultaneously evaluating miRNA dysregulation across different cell types in two well‐known AIDs, namely, MS and T1D. Specifically, we chose to study three immune populations, namely, CD14^+^ monocytes, neutrophils, and CD8^+^ T cells, as they are the most common infiltrates detected in MS brains and in the T1D pancreatic tissue.

Simultaneous analysis of three different leukocyte populations allowed characterization of the differential impact of miRNA dysregulation. For example, in CD8^+^ T cells from T1D subjects, the reduced expression of Tc17‐promoting miRNAs suggests a lower peripheral frequency of Tc17 cells within the analyzed cohort. This finding is consistent with the dynamics of Tc17 cells in T1D, which expand at onset but decrease in individuals with longer disease duration [26, 60]. The possibility that Tc17 cells mobilize to pancreatic islets, leading to reduced peripheral abundance, warrants further investigation. In addition, miRNA dysregulation in this cell type may increase T cell activation and survival by inducing ABCA1‐mediated cholesterol efflux [13]. Besides lymphocytes, our findings suggest that miRNA dysregulation in T1D monocytes contributes to the promotion of M1 polarization, induction of diabetic vascular damage, and increased cell proliferation. In neutrophils, no functional categories were associated with miRNA dysregulation. This may reflect their reliance on rapid, posttranscriptional effector mechanisms such as degranulation and NETosis, rather than on slower miRNA‐mediated regulation. Alternatively, it may result from methodological factors, including interpatient variability or broadly distributed regulatory effects. Larger cohorts or alternative analyses may be needed to reveal subtle miRNA roles in this cell type.

Conversely, miRNA impairment in CD8^+^ T cells from RRMS patients suggests a potential mechanism for IL‐17 secretion, strengthening the link between Tc17 cells and MS activity [21]. Furthermore, the observed reduction in miR‐15a‐5p and miR‐16‐5p expression is indicative of CD8^+^ Treg expansion [22] and reduced sensitivity to the apoptotic process. MiRNAs in CD8^+^ T cells are also involved in the maintenance of a promigratory phenotype, which may be involved in CNS trafficking. On the other hand, neutrophils from RRMS patients show pronounced alterations in ROS production, NET formation, survival, and motility, although the role of miRNA dysregulation in this cell type remains unclear.

Due to the unavailability of suitable omics datasets for CD8^+^ T cells, CD14^+^ monocytes, and neutrophils in T1D patients, the integrative analysis was only feasible for MS. Specifically, given the relevance of the identified miRNA regulatory networks in CD8^+^ T cells from MS patients, our integrative analysis focused on this immune cell population. In particular, we demonstrated that the transcriptional dysregulations in this cell type involve the same biological processes regulated by DE‐miRNAs (Figure 3C). Moreover, we showed that miRNAs exert a significant influence on gene expression (Figure 3H) and may play a role in promoting Tc17 differentiation, CD8^+^ Treg differentiation, apoptosis regulation, and T cell migration (Figure 3I). In support of this idea, we demonstrated that the transcriptional activity of FOXP3, RORC, and IRF4 is significantly increased in MS patients compared with controls (Figure 5H–J). In this context, we also reported that the frequency of CD8^+^ Tregs and Tc17 cells in the peripheral blood of MS patients is almost doubled (Figure 5K,L). Given the importance of CD8^+^ Tregs in autoimmunity, we experimentally validated the overexpression of FOXP3 (Figure 4G) and provided evidence for a negative correlation between FOXP3 expression and miR‐15a‐5p (Figure 4M). Analogously, we also observed that BCL2 is overexpressed in MS patients (Figure 4F) and that its levels are negatively correlated with those of miR‐155‐5p and miR‐15a‐5p (Figure 4K,L), further suggesting the impact of miRNA dysregulation on apoptosis regulation.

The mechanisms whereby miRNAs may orchestrate CD8^+^ T cell commitment take place at different levels. For example, transcriptomic analysis has shown that IRF4 is overexpressed in CD8^+^ T cells from MS patients. Notably, IRF4 expression is known to be negatively regulated by miR‐15a‐5p, miR‐16‐5p, and miR‐155‐5p [61], which are downregulated in CD8^+^ T cells. The potential consequences of this regulatory imbalance could lead to an increased differentiation of Tc17 cells [62], but also to an increased frequency of effector Tregs [23, 63, 64]. In support of this notion, it has been shown that the depletion of miR‐15‐5p and miR‐16‐5p is associated with increased effector Treg frequencies due to the absence of IRF4 inhibition [22]. Furthermore, knockdown of miR‐15a‐5p and miR‐16‐5p resulted in increased FOXP3 expression and improved suppression of allogeneic lymphocyte proliferation [47]. Additionally, our results suggest that downregulation of miR‐629‐5p and miR‐6849‐5p drives the observed overexpression of IL‐2Rβ, thereby supporting IL‐2 signaling that stimulates FOXP3 production via the JAK1/3–STAT5 pathway. Moreover, TGFBR1 production may be influenced by several downregulated miRNAs, including miR‐152‐3p and miR‐155‐5p. This is of particular importance given the indispensable role of TGF‐β signaling in both differentiation programs. Regarding Tc17 differentiation, the reduced expression of miR‐155‐5p is able to increase the activity of STAT3 (Figure S7C), which is required for RORγt production [65]. In this context, the downregulation of mir‐152‐3p and miR‐155‐5p may also promote Tc17 differentiation by acting on the production of the IL‐6 receptor complex, the activation of which is required for RORγt expression via the JAK–STAT3 pathway [65]. In addition, miR‐152‐3p and miR‐146a‐5p may also regulate the production of the IL‐23 receptor [66, 67], another receptor involved in Tc17 differentiation. Notably, miR‐146a‐5p is also predicted to bind directly to the promoter of IL17A, although experimental validation is necessary to substantiate this hypothesis.

**FIGURE 6:**
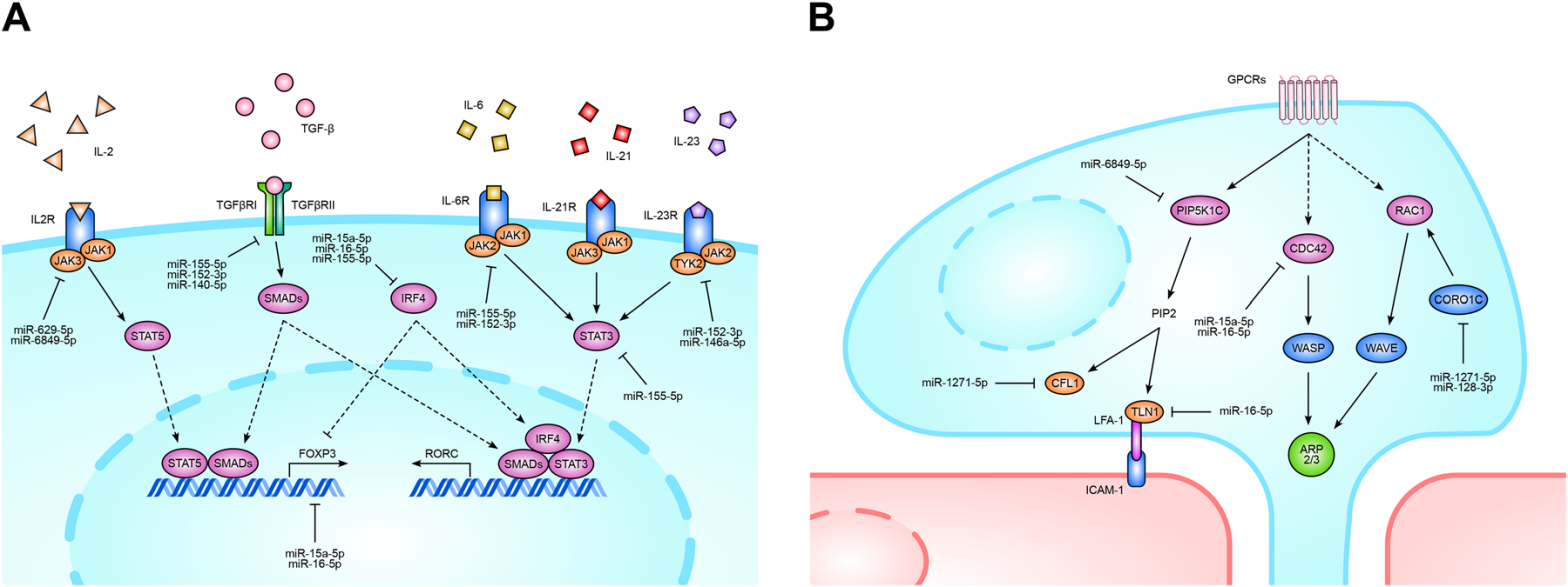
The compromised miRNA regulatory networks in MS‐derived CD8^+^ T cells. Panel (A) illustrates the perturbed miRNA–target interactions that promote CD8^+^ Treg and Tc17 differentiation. Panel (B) shows the molecular dysregulations through which miRNAs induce a promigratory phenotype in CD8^+^ T cells.

Apart from their role in Tc17/CD8^+^ Treg differentiation, miRNAs are also involved in the regulation of lymphocyte migration. For example, downregulation of miR‐15a‐5p and miR‐16‐5p may play a specific role in coordinating directional movement through their control of CDC42, a critical GTPase that localizes to the leading edge and promotes actin polymerization through the WASP–Arp2/3 pathway [68]. This is further supported by the increased expression of CDC42 in MS patients. Additionally, the downregulation of miR‐16‐5p may also be responsible for the overexpression of talin‐1 [69], a protein that stabilizes integrins and promotes their activation. We also observed the overexpression of PIP5K1C, an enzyme responsible for the production of PIP2, which in turn mediates the activation of talin‐1 and cofilin‐1. Notably, PIP5K1C was identified as a predicted target of miR‐6849‐5p. Moreover, the downregulation of miR‐1271‐5p and miR‐128‐3p may explain the upregulation of cofilin‐1, which is involved in actin turnover required for sustained movement [70], and coronin 1C, a protein that maintains cell polarization by regulating the activation and subcellular location of RAC1 [71]. Finally, the downregulation of miR‐146a‐5p may explain the increase of MMP9 [72], thereby promoting the degradation of the extracellular matrix and the invasiveness of CD8^+^ T cells [73]. Regarding cell survival, decreased expression of miR‐15a‐5p, miR‐16‐5p, and miR‐155‐5p is likely responsible for the reduced sensitivity of CD8^+^ T cells to apoptotic stimuli due to lack of BCL2 inhibition [18, 31, 74]. The intricate network of miRNA–target interactions that governs CD8^+^ Treg and Tc17 differentiation is illustrated in Figure 6A. Conversely, Figure 6B illustrates a summary of miRNA‐mediated mechanisms involved in the regulation of CD8^+^ T cell migration.

In recent years, the contribution of Tc17 cells in MS has become clear. Increased frequencies of Tc17 cells have been observed in both CSF and blood of MS patients [21, 27], as well as in active areas of MS lesions [27]. Furthermore, Lückel et al. showed that dimethyl fumarate reduced Tc17 cells only in patients who responded to treatment [75]. Regarding CD8^+^ Tregs, Houston et al. demonstrated an increase in CD8+ Tregs after fingolimod therapy [76]. In contrast, Frisullo et al. did not observe differences between patients and controls, but did observe a marked increase in CD8^+^ Tregs between MS patients in remission and those in relapse [77]. Taken together, the expansion of CD8^+^ Tregs and Tc17 cells may indicate a subtle balance between disease‐promoting and compensatory mechanisms. If true, this may open the way to the development of novel therapeutic options designed to induce this protective mechanism. However, it will be of interest to evaluate whether this mechanism is active in subgroups of MS patients or whether it is specific of the remission phase of the disease. Interestingly, the clinical phenotype of the cohort analyzed in this study was characterized by a stable disease course, a low EDSS score and the absence of active immunomodulating therapy (Table 1), suggesting that a positive, compensatory mechanism may be active in this cohort.

To place our findings in the context of existing knowledge, we compared them with previous studies investigating miRNA dysregulation in MS and T1D. Earlier reports identified altered levels of miR‐155‐5p, miR‐146a‐5p, and members of the miR‐15/16 cluster in bulk PBMCs or CD4^+^ T cells from RRMS patients [78, 79], and similar alterations have been linked to T‐cell‐mediated β‐cell destruction in T1D [80, 81]. However, little is currently known about how miRNA networks operate within ex vivo CD8^+^ T cells, CD14^+^ monocytes, and neutrophils. In this concern, most prior work has either neglected these subsets, or focused on a small number of miRNAs (e.g., Amoruso et al. [82]), and no genome‐wide analyses have been performed in these populations. Our results further reveal both conserved and disease‐specific mechanisms. The downregulation of miR‐15a‐5p and miR‐16‐5p in CD8^+^ T cells leads to BCL2 overexpression, supporting a conserved antiapoptotic mechanism previously observed in other autoimmune and cancer contexts [31, 79, 83]. In contrast, miR‐155‐5p displays a distinct pattern potentially specific to MS: while it is typically upregulated in T1D and other inflammatory settings [80, 84], we found it significantly downregulated in CD8^+^ T cells from RRMS patients. This observation aligns with Elkhodiry et al., who reported similar downregulation and associations with EDSS scores and disease duration [85]. Such reduction may relieve repression of STAT3, the IL‐6 receptor complex, and TGFBR1, thereby promoting Tc17 and CD8^+^ Treg differentiation—an immunoregulatory axis central to MS but less prominently described in T1D. Finally, we identified downregulation of miR‐152‐3p as a novel regulatory mechanism affecting IL‐6 and IL‐23 receptor expression. Although this miRNA has been implicated in other diseases [86], its potential role in modulating Tc17 differentiation in MS has not been previously reported.

In considering the limitations of our study, it is important to note that our analyses are based on observational multi‐omics data and therefore primarily capture associations rather than causal relationships. As such, the proposed framework should be regarded as hypothesis‐generating, designed to identify the most promising regulatory candidates for subsequent experimental investigation. To support our computational findings, we performed a preliminary validation of five key regulatory miRNAs, confirming their differential expression and the expected inverse regulation of their target genes in ex vivo samples from RRMS patients (Figure 4). This validation provides biological support for the inferred interactions and reinforces their potential relevance in disease contexts. Nevertheless, while our integrative analyses point to a central role for these miRNAs in immune regulatory pathways, functional assays—such as miRNA overexpression or knockdown experiments in cellular or animal models—will be essential to demonstrate causality and to elucidate the precise mechanistic contribution of these regulators to MS pathogenesis. These studies represent the next necessary step toward translating our computational predictions into experimentally validated insights.

Last, the cell‐type‐specific multi‐omics framework presented here provides a transferable analytical paradigm suitable for diverse disease contexts as multi‐omics data become increasingly available—a trend accelerating across autoimmune and chronic disorders [87]. For instance, our pipeline could be applied to uncover cell‐specific mechanisms in other autoimmune conditions, such as thyroid eye disease, where multi‐omics approaches are already advancing biomarker discovery [88]. Likewise, it could prove valuable in nonautoimmune settings, such as metabolic dysfunction‐associated steatohepatitis, where single‐cell multi‐omics is illuminating key cellular drivers of disease progression [89]. Overall, this framework establishes a versatile template for high‐resolution, cell‐specific translational research across a wide spectrum of diseases enriched with multi‐omics data.

In conclusion, we have provided evidence for altered expression of miRNAs in CD8^+^ T cells in MS and T1D, highlighting the importance of this novel class of genes in the regulation of immune protection as well as pathogenic responses. The integration of different multiparameter technologies revealed not only the information of a single regulatory layer, but also the interplay between different molecular entities. The clear future direction of this work is to clinically validate these miRNA signatures in a large, prospective patient cohort to establish their utility as biomarkers for disease course and therapeutic response. A new understanding of the key mechanisms underlying disease progression will potentially have important and beneficial implications for clinical care, treatment targets, and regulatory decision‐making.

## 4 Materials and Methods

### 4.1 Study Cohort and Recruitment

The study protocol was approved by the ethics committees of HSG (Monza, Italy) and HSR (Milan, Italy), and all participants provided written informed consent prior to inclusion. A total of 61 subjects were enrolled and divided into two independent cohorts based on the recruiting center.

The first cohort was recruited at HSG and comprised 20 RRMS patients and 15 age‐matched HCs. The second cohort was enrolled at HSR and included 12 patients with T1D and 14 age‐matched HCs. An overview of the study cohort and clinical characteristics is provided in Table 1.

### 4.2 Harvesting and Enrichment of Immune Cells

Blood samples were collected from all MS/T1D patients and HCs from the antecubital vein into a K_2_EDTA‐coated vacutainer using a 19G needle to prevent neutrophil activation. PBMCs were subsequently obtained by Ficoll‐Paque centrifugation. Monocytes and CD8^+^ T cells were isolated by positive selection using magnetic beads (Miltenyi Biotec, Bergisch Gladbach, Germany). The purity of the obtained cell subsets was checked by flow cytometry and found to be >90%. Neutrophil isolation was performed according to a modified Miltenyi Biotech isolation kit protocol. The blood was centrifuged, the enriched plasma was discharged, and the neutrophils were subjected to negative selection using a dedicated Miltenyi magnetic beads system. A supplementary red blood cell lysis was performed according to the instructions of the supplier. The purity of the neutrophil suspension was determined to be at least 90% by means of flow cytometry analysis.

### 4.3 RNA Isolation

The mirVana RNA isolation kit (Ambion, Inc., Austin, TX, USA) was used to extract total RNA from CD14^+^ monocytes, CD8^+^ T cells, and neutrophils, including small noncoding RNAs, in accordance with the manufacturer's protocol. The quantity of RNA was determined using a ND‐1000 spectrophotometer (Nanodrop Technology, Cambridge, UK), and the integrity of the RNA was evaluated using the Agilent 2100 Bioanalyzer (Agilent Technologies, Santa Clara, CA, USA). The 260/280 ratio was between 1.7 and 2.1, and the RINs (RNA integrity numbers) were >6 for all samples.

### 4.4 Microarray Processing

For the analysis of miRNA expression, we labeled 1 µg of total RNA with biotin using the FlashTag HSR RNA labeling kit for Affymetrix miRNA arrays (Genisphere LLC., Hatfield, PA, USA), following the manufacturer's instructions. The labeled RNA was then hybridized to Affymetrix GeneChip miRNA 4.1 microarrays, also following the manufacturer's instructions.

### 4.5 MiRNA Microarray Data Analysis

Raw CEL files were imported into the R environment [90] and underwent normalization using the robust multiarray average algorithm implemented in the oligo R package [91]. Then, expression data were transformed in log_2_ scale and advanced quality control was performed through the arrayQualityMetrics [92] package. Samples flagged as outliers were excluded from downstream analyses. The probesets were annotated according to the annotations provided by Affymetrix, and filtered to retain only the probes targeting human miRNAs. Since the majority of annotated miRNAs are expressed at relatively low levels [93], to reduce the burden of multiple testing, only the 400 most highly expressed miRNAs were considered for CD8^+^ T cells and neutrophils, while the 500 most highly expressed miRNAs were considered for monocytes. MDS plots and linear mixed models, as implemented in the variancePartition package [94], were used to identify the sources of biological and technical variability. Furthermore, we estimated surrogate variables through the SVA package [95] to account for unwanted sources of variation. Array quality weights were computed in limma [96] and included in the model to account for differences between arrays. DE‐miRNAs were identified for each comparison by a moderated *t*‐test, as implemented in the eBayes function from the limma package. The Storey's *q*‐value approach [97] was used to adjust *p* values for multiple testing.

### 4.6 MiRNA Functional Enrichment

After differential expression analysis, the DE‐miRNAs identified in each cell type were used to perform an overrepresentation analysis based on the manually curated miRNA categories annotated in the TAM 2.0 database [98] (http://www.lirmed.com/tam2/). Only miRNA sets with a minimum of eight miRNAs were considered. *p* Values were calculated using the hypergeometric distribution and subsequently adjusted for multiple testing through the Benjamini–Hochberg approach. Terms with an adjusted *p* value of less than 0.1 were deemed statistically significant.

### 4.7 CD8^+^ Transcriptome Analysis

The transcriptomic changes of CD8^+^ T cells in MS were assessed using a publicly available dataset that was retrieved from GEO (accession number: GSE32988). Only raw data concerning CD8^+^ T cell expression in RRMS patients before transplantation and in HCs were used. The raw files were loaded into R, and the intensities were background corrected and quantile normalized. Additionally, the expression of probes targeting the same gene was averaged using the avereps function, and probes with low expression, defined as normalized intensity values lower than 6, were discarded. MDS plots were generated to assess data variability, and a linear model was fitted to the expression data. Finally, array quality weights were estimated, and differential expression was performed through a moderated *t*‐test. *p* Values were corrected using the Storey's *q*‐value method, and genes with *q* < 0.1 and absolute fold change > 1.5 were defined as DEGs. The GSEA algorithm was used to conduct functional enrichment analysis with the gene‐sets provided by GO. The *t* statistic returned from differential expression analysis was used to rank genes in descending order. Statistically significant categories were identified as those with Benjamini–Hochberg adjusted *p* values less than 0.1.

### 4.8 MiRNA Activity Estimation

To estimate miRNA activity based on the differential expression of their target genes, the enrichMiR software [99] was used (https://ethz‐ins.org/enrichmir/). MiRNA binding sites were collected from the TargetScan v8 database [100], with the exclusion of binding sites involving miRNAs that were not measured in the present experiment. Subsequently, the overrepresentation of each binding site in upregulated and downregulated genes (Storey's *q* value < 0.1) was calculated using the Fisher's exact test. The resulting *p* values were then corrected for multiple testing through the Benjamini–Hochberg approach, and binding sites with an adjusted *p* value < 0.05 were deemed significant. Finally, miRNAs with significant estimated activity were intersected with the observed DE‐miRNAs. To identify target genes, validated miRNA–target interactions were retrieved from the miRTarBase 2025 database [45], and high‐confidence predicted interactions were collected from the miRNA Data Integration Portal (mirDIP) database [46]. Then, an overrepresentation analysis was performed according to the gene‐sets defined in GO (Benjamini–Hochberg adjusted *p* value < 0.1).

### 4.9 RT‐qPCR Analysis of miRNAs and Target Genes

To detect miRNA in human cells, 1 µg of total RNA was used for the synthesis of cDNA, employing the QuantiMir cDNA Kit (System Biosciences, Mountain View, CA), in accordance with the manufacturer's protocol. MiRNA primers were designed based on sequences from the miRBase database [101] (https://www.mirbase.org/) using primer3 software [102]. The primers were designed to have a melting temperature between 57 and 62°C. Additionally, primer sequences with stable homodimerization or hairpin structures (Δ*G* < −9 kcal/mol) were edited. Then, all generated primer sequences were subjected to BLAST verification to ensure specificity for the designated target miRNA. The amplification of target sequences was verified by analyzing melting curve peaks of all miRNA primers on a THP‐1 cell line.

For target gene analysis, cDNA was prepared using the High Capacity cDNA Reverse Transcription Kits (Applied Biosystems) and the primer sequences reported in Table S10. Quantitative analysis of gene expression, as well as endogenous controls, was performed using TaqMan assays. To ensure equal amplification efficiencies for the target and reference genes, validation experiments were performed using a series of cDNA dilutions spanning several orders of magnitude. The resulting Ct values were plotted against log_10_[cDNA quantity], and reaction efficiencies (E%) were calculated using the formula: E%=[−1+10(−1/slope)]×100. Only primer pairs producing a linear regression with $R^{2}>0.99$ were used in subsequent experiments.

The relative expression values were then calculated using the $2^{-\Delta \Delta Ct}$ method. Prior to conducting the statistical analysis, evident deviations of ΔCt values from the normal distribution were excluded through *Q*–*Q* plots and the Shapiro–Wilk test (*α* = 0.05). A two‐sample Welch's *t*‐test was employed to compare the mean ΔCt values between patients and controls (*p* < 0.05). Pearson's correlation was used to measure the correlation between the ΔCt values of selected miRNA–target pairs.

### 4.10 Single‐Cell Analysis of CD8^+^ T Cells in RRMS

Raw single‐cell transcriptomes were obtained from a public dataset (accession number: GSE138266). PBMCs were imported into R and CD8^+^ T cells were isolated based on the simultaneous expression of both CD3G and one of CD8A and CD8B. Low‐quality cells, characterized by a limited number of total reads, a small number of detected genes, or a high percentage of reads mapping to mitochondrial DNA, were removed based on the perCellQCFilters function from the scuttle package [103]. The expression matrix was normalized for the library size of each cell and transformed in log_2_ scale. Later, the mean‐variance trend was modeled for each sample, and the 5000 most variable genes were identified. Single‐cell transcriptomes from distinct donors underwent batch correction through the mutual nearest neighbors correction algorithm [104]. UMAP dimensionality reduction was then performed over the space of corrected principal components. To identify the various CD8^+^ T cell subsets, a shared nearest‐neighbor graph was constructed on top of the corrected dimensions based on the five nearest neighbors of each cell. The Jaccard index was used to weight the edges. Clusters were then identified using the Louvain community detection algorithm. The SingleR method [59] was used to assign cell labels based on the Novershtern dataset [105] as reference. To ensure the consistency of the assignment, the cell annotation was repeated using the Monaco dataset as a reference [106]. Furthermore, marker genes of each cluster were identified, and cluster identity was manually refined. Contaminant clusters consisting of myeloid cells were removed.

### 4.11 Pseudobulk Differential Expression Analysis of CD8^+^ T Cells

The aggregateAcrossCells function from the scuttle package was used to aggregate single cell counts for each individual. Genes with low expression levels were filtered, and normalization factors were calculated using the trimmed mean of *M* values method as implemented in the calcNormFactors function from edgeR [107]. After modeling the mean‐variance trend via estimation of negative binomial dispersions, a negative binomial generalized linear model was fitted for each gene, and the quasi‐likelihood (QL) *F* test was used to compare gene expression between RRMS patients and controls. *p* Values were then corrected with the Storey's *q*‐value approach, and genes with *q* values less than 0.1 were considered significant. GSEA was performed as previously described using genes ranked in descending order based on this formula: $-\mathrm{lo}g_{10}p\cdot \mathrm{sign}(\mathrm{lo}g_{2}\mathrm{FC})$.

### 4.12 Transcription Factor Activity Estimation From Single‐Cell Data

Transcription factor (TF)—gene interactions were defined according to the CollecTRI network [108]. Multivariate decision trees, as implemented in the run_mdt function from the decoupleR package [57], were employed to estimate TF activities for each cell. The activity of each TF was averaged across CD8^+^ T cells from each donor. The normality of TF activities was assessed using Q‐Q plots and the Shapiro–Wilk test, and differences between RRMS patients and HCs were evaluated using the Welch's *t*‐test. *p* Values lower than 0.05 were deemed to be statistically significant.

### 4.13 Differential Abundance of CD8^+^ Tregs and Tc17 Cells

For the identification of CD8^+^ Tregs, a curated signature comprising FOXP3, IKZF2, PRDM1, CTLA4, PDCD1, LAG3, IL10, TGFB1, IL2RA, ENTPD1, NT5E, IKZF4, GZMB, and ENPP1 was used. Based on the distribution of AUC scores and the anticipated frequency of CD8^+^ Tregs in the peripheral blood [109], a threshold of 0.17 was selected. Conversely, to identify Tc17 cells, a molecular signature comprising RORC, BATF, STAT3, IL17A, IL17F, IL22, IL23R, and KLRB1 (CD161) was used. In this case, a threshold of 0.22 was chosen on the basis of the expected frequency of Tc17 cells [109]. To verify the accuracy of cell assignments, marker genes were defined for both CD8^+^ Tregs and Tc17 cells using the scoreMarkers function. Subsequently, an overrepresentation analysis was conducted using the top 250 marker genes, selected by decreasing mean AUC, to assess the enrichment of immune regulatory processes for CD8^+^ Tregs and IL‐17‐related pathways for Tc17 cells. Following their identification, the fraction of CD8^+^ Tregs and Tc17 cells was compared between RRMS patients and HCs. After assessing data normality through *Q*–*Q* plots and the Shapiro–Wilk test, the Welch's *t*‐test was then employed to ascertain the statistical significance of the observed differences, with a *p*‐value threshold of 0.05.

## Author Contributions

*Research design*: Maria Foti, Manuela Battaglia, Giovanni Malerba, and Roberta Rigolio. *Experimental operations*: Jacopo Ronchi, Roberta Rigolio, Davide Trevisan, Angela Papagna, Angela Stabilini, and Martina Gallinaro. *Guidance on clinical patient evaluation*: Maria Letizia Fusco, Martina Gaia Cogo, and Guido Cavaletti. *Analysis of results and visualization*: Jacopo Ronchi and Maria Foti. *Writing*: Maria Foti and Jacopo Ronchi. All authors have read and approved the final manuscript.

## Funding

This work was supported by the Italian Ministry of Health (Grant Number. RF‐2016‐02364384); Cariplo Foundation 2013 (N.RF.2013‐0941); Italian Foundation for Multiple Sclerosis (FISM2009/R/10); Italian Foundation for Multiple Sclerosis (FISM 2009/R/22); and Italian Ministry of Education and Research (PRIN‐COFIN20077NFBH8_003).

## Ethics Statement

This study was approved by the ethics committee of San Gerardo Hospital (No. C2013‐0941) and ethics committees of other study sites. Informed consent was obtained from all participants.

## Conflicts of Interest

Authors Maria Letizia Fusco, Martina Gaia Cogo, and Guido Cavaletti are members of Fondazione IRCCS San Gerardo dei Tintori, but have no conflicts of interest to disclose. The other authors declare no conflicts of interest.

## Supporting information



Figure S1: MDS plots of miRNA expression before batch‐effect correction. Panels (A)–(C) show the variability of miRNA expression in CD8^+^ T cells, CD14^+^ monocytes, and neutrophils from MS patients. Similarly, panels (D)–(F) show the variability for CD8^+^ T cells, CD14^+^ monocytes, and neutrophils for the T1D cohort.
Figure S2: MDS plots of miRNA expression after batch‐effect correction. Panels (A)–(C) illustrate that after adjustment for batch effects, miRNA expression is mainly dominated by differences between MS patients and controls for both CD8^+^ T cells, CD14^+^ monocytes, and neutrophils. In the same way, the corrected miRNA expression profiles show disease‐related differences between CD8^+^ cells, CD14^+^ monocytes, and neutrophils for T1D, as evidenced in panels (D)–(F).
Figure S3: MiRNA functional enrichment analysis in MS and T1D. The panel shows the enriched categories resulting from the overrepresentation analysis of differentially expressed miRNAs in each cell type and for each comparison. The *y*‐axis represents the negative logarithm of the Benjamini–Hochberg adjusted *p* value, while the *x*‐axis shows the *z*‐score of the enrichment, defined as $\frac{u-d}{\sqrt{n}}$, where *u* is the number of upregulated miRNAs belonging to the category, *d* is the number of downregulated miRNAs belonging to the category, and *n* is the total size of the category.
Figure S4: Preprocessing of CD8^+^ transcriptomes. Panel (A) shows the density of median probe intensities for each gene. To remove the majority of lowly expressed genes, we used a cutoff value of 6. Panel (B) illustrates that gene expression of CD8^+^ T cells in the MDS space is mainly affected by disease state.
Figure S5: Influential miRNA–target interactions in CD8^+^ T cells from RRMS patients. Panel (A) shows influential pairs associated with Treg differentiation and IL‐10 production. Panel (B) shows influential pairs linked to leukocyte chemotaxis. Panel (C) shows influential pairs implicated in Tc17 differentiation and IL‐17 secretion. Panel (D) shows influential pairs involved in the negative regulation of apoptotic pathways.
Figure S6: Cluster annotation of single‐cell CD8^+^ transcriptomes. Panel (A) displays the score of cluster identity predictions according to the SingleR method using the Novershtern reference. Panel (B) shows the expression of the top marker genes for each cluster.
Figure S7: Pseudobulk analysis and identification of Tc17 cells and CD8^+^ Tregs. Panel (A) shows the variability of gene expression in pseudobulks of CD8^+^ T cells. Panel (B) represents DEGs derived from differential expression analysis of pseudobulks between MS patients and controls. Panels (C) and (D) show the overactivation of STAT3 and RUNX1, respectively, based on estimated transcriptional activities. Panel (E) shows the enrichment of IL‐17‐related categories in the identified Tc17 population. Panel (F) shows the enrichment of regulatory categories in the identified CD8^+^ Treg subset.
Table S1 Excluded samples for each cell type and condition.
Tables S2–S4 contain the lists of differentially expressed miRNAs identified in CD8^+^ T cells, CD14^+^ monocytes, and neutrophils from MS patients.
Tables S5–S7 contain the lists of differentially expressed miRNAs identified in CD8^+^ T cells, CD14^+^ monocytes, and neutrophils from T1D patients.
Table S8 lists the DEGs identified in CD8^+^ T cells from MS patients.
Table S9 lists the DEGs identified through pseudobulk differential expression analysis of CD8^+^ T cells from MS patients.
Table S10 contains the primer sequences used for the RT‐qPCR experiments presented in the manuscript.

Supporting File 1: mco270647‐sup‐0002‐tableS2.csv

Supporting File 2: mco270647‐sup‐0003‐tableS3.csv

Supporting File 3: mco270647‐sup‐0004‐tableS4.csv

Supporting File 4: mco270647‐sup‐0005‐tableS5.csv

Supporting File 5: mco270647‐sup‐0006‐tableS6.csv

Supporting File 6: mco270647‐sup‐0007‐tableS7.csv

Supporting File 7: mco270647‐sup‐0008‐tableS8.csv

Supporting File 8: mco270647‐sup‐0009‐tableS9.csv

Supporting File 9: mco270647‐sup‐0010‐tableS10.csv

## Data Availability

The miRNA microarray data of patients with T1D, MS, and healthy volunteers have been deposited in the GEO database, and are publicly available under accession number GSE289530. For the analysis of CD8^+^ bulk transcriptome in MS, the GSE32988 dataset from GEO has been used. Conversely, for the single‐cell analysis, the GSE138266 dataset from GEO was used. All R scripts used to generate the findings and figures presented in this manuscript have been made publicly available on GitHub (https://github.com/jacopo‐ronchi/ms_t1d_mirna_analysis).
