# Supplementary material for: Multi‐Omics Analysis in Autoimmunity: Identification of MicroRNA Regulatory Networks and Cell‐Type‐Specific Dysregulations in Multiple Sclerosis and Type 1 Diabetes
Source: MedComm (2020). 2026 Apr 2;7(4):e70647. doi: 10.1002/mco2.70647 (PMC13051886; doi:10.1002/mco2.70647)
Supplement: Supplementary file 1 — Figure S1: MDS plots of miRNA expression before batch‐effect correction. Panels (A)–(C) show the variability of miRNA expression in CD8+ T cells, CD14+ monocytes, and neutrophils from MS patients. Similarly, panels (D)–(F) show the variability for CD8+ T cells, CD14+ monocytes, and neutrophils for the T1D cohort. Figure S2: MDS plots of miRNA expression after batch‐effect correction. Panels (A)–(C) illustrate that after adjustment for batch effects, miRNA expression is mainly dominated by differences between MS patients and controls for both CD8+ T cells, CD14+ monocytes, and neutrophils. In the same way, the corrected miRNA expression profiles show disease‐related differences between CD8+ cells, CD14+ monocytes, and neutrophils for T1D, as evidenced in panels (D)–(F). Figure S3: MiRNA functional enrichment analysis in MS and T1D. The panel shows the enriched categories resulting from the overrepresentation analysis of differentially expressed miRNAs in each cell type and for each comparison. The y‐axis represents the negative logarithm of the Benjamini–Hochberg adjusted p value, while the x‐axis shows the z‐score of the enrichment, defined as u−dn, where u is the number of upregulated miRNAs belonging to the category, d is the number of downregulated miRNAs belonging to the category, and n is the total size of the category. Figure S4: Preprocessing of CD8+ transcriptomes. Panel (A) shows the density of median probe intensities for each gene. To remove the majority of lowly expressed genes, we used a cutoff value of 6. Panel (B) illustrates that gene expression of CD8+ T cells in the MDS space is mainly affected by disease state. Figure S5: Influential miRNA–target interactions in CD8+ T cells from RRMS patients. Panel (A) shows influential pairs associated with Treg differentiation and IL‐10 production. Panel (B) shows influential pairs linked to leukocyte chemotaxis. Panel (C) shows influential pairs implicated in Tc17 differentiation and IL‐17 secretion. Panel (D [file MCO2-7-e70647-s007.pdf]

Multi-Omics Analysis in Autoimmunity: Identification of MicroRNA Regulatory Networks and Cell-Type-Specific Dysregulations in Multiple Sclerosis and Type I Diabetes

Jacopo Ronchi[#1,6], Roberta Rigolio[#1], Davide Maria Trevisan[‡1], Angela Papagna[1], Angela Stabilini[2], Martina Gallinaro[3], Maria Letizia Fusco[4], Martina Gaia Cogo[§4], Guido Cavaletti[1,4], Giovanni Malerba[3], Manuela Battaglia[2], and Maria Foti[*1,5]

[1]School of Medicine and Surgery, University of Milano-Bicocca, Monza (MB), Italy
[2]Diabetes Research Institute, IRCCS San Raffaele, Scientific Institute, Milan, Italy
[3]Department of Neurosciences, Biomedicine and Movement Sciences, University of Verona, Italy
[4]Fondazione IRCCS San Gerardo dei Tintori, Monza, Italy
[5]BicOMICs, University of Milano-Bicocca, Monza (MB), Italy
[6]PhD Program in Neuroscience, Medicine and Surgery Department, University of Milano-Bicocca, Monza, Italy

#: These authors contributed equally to the study.
∗: This author has the corresponding authorship. Send correspondence to: maria.foti@unimib.it

Present address:
‡: Ebba Biotech AB, Nobels väg 16, S-171 65 Solna, Sweden.
§: Hospital Pio XI of Desio, ASST-Brianza.

Supplementary Information

Supplementary Figures

Figure S1 – MDS plots of miRNA expression before batch-effect correction 2
Figure S2 – MDS plots of miRNA expression after batch-effect correction 3
Figure S3 – MiRNA functional enrichment analysis in MS and T1D 4
Figure S4 – Pre-processing of CD8$^+$ transcriptomes 5
Figure S5 – Influential miRNA–target interactions in CD8$^+$ T cells from RRMS patients 6
Figure S6 – Cluster annotation of single-cell CD8$^+$ transcriptomes 7
Figure S7 – Pseudobulk analysis and identification of Tc17 cells and CD8$^+$ Tregs . . . 8

Supplementary Tables

Table S1 – Excluded samples for each cell type and condition 9
Table S2–S10 - Online Material 10

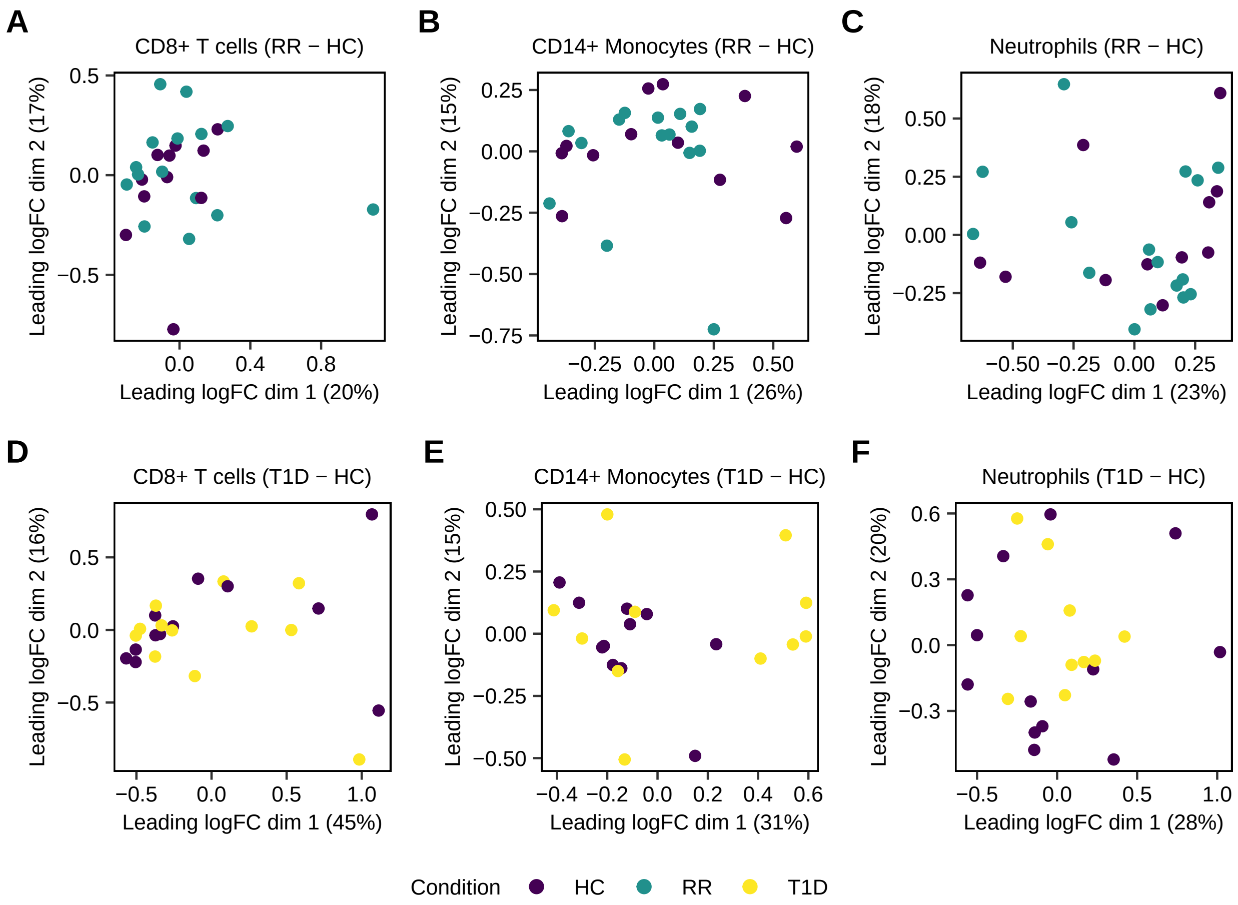

Figure S1: MDS plots of miRNA expression before batch-effect correction. A-C show the variability of miRNA expression in CD8$^+$ T cells, CD14$^+$ monocytes and neutrophils from MS patients. Similarly, **D-F** show the variability for CD8$^+$ T cells, CD14$^+$ monocytes and neutrophils for the T1D cohort.

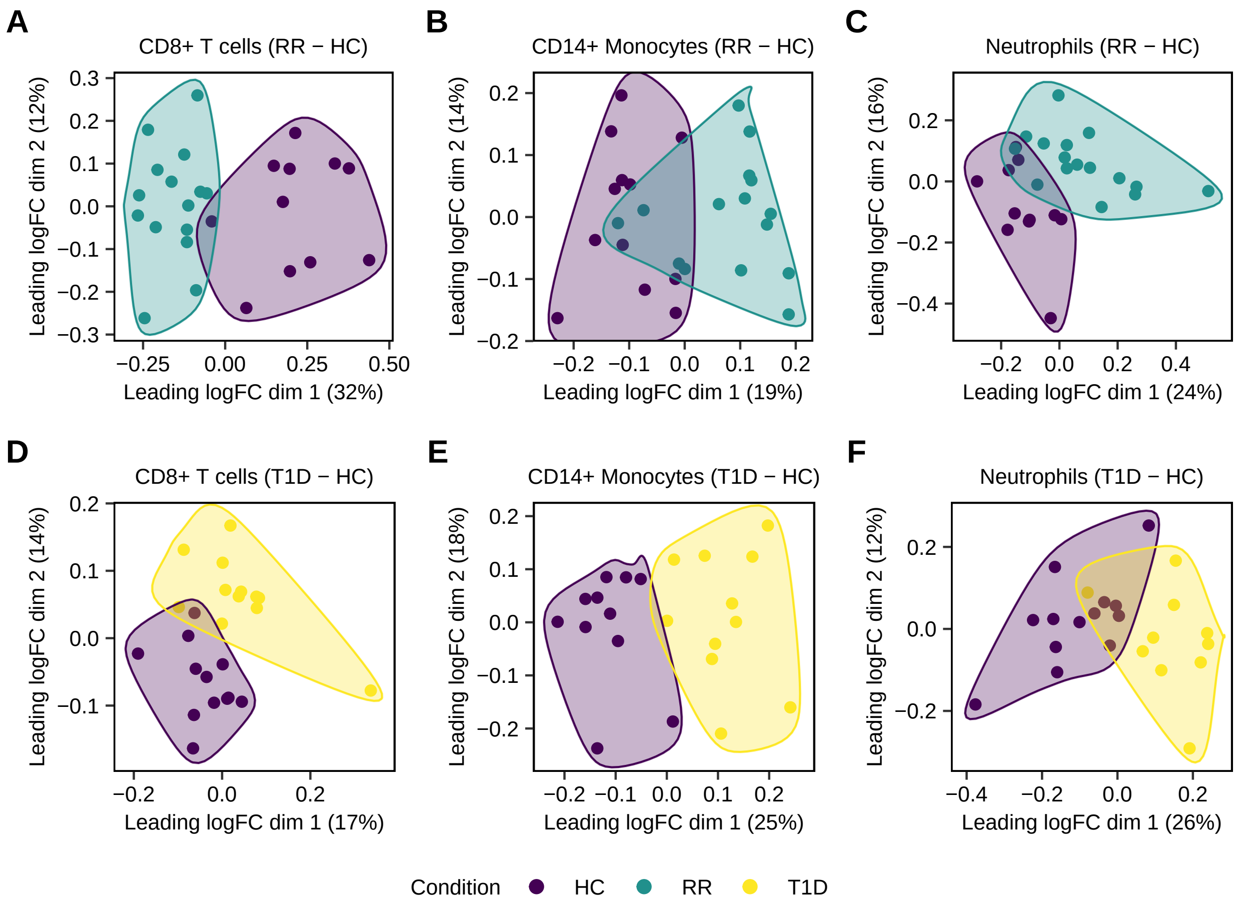

Figure S2: MDS plots of miRNA expression after batch-effect correction. **A-C** illustrate that after adjustment for batch effects, miRNA expression is mainly dominated by differences between MS patients and controls for both CD8^{+} T cells, CD14^{+} monocytes and neutrophils. In the same way, the corrected miRNA expression profiles show disease-related differences between CD8^{+} cells, CD14^{+} monocytes and neutrophils for T1D, as evidenced in **D-F**.

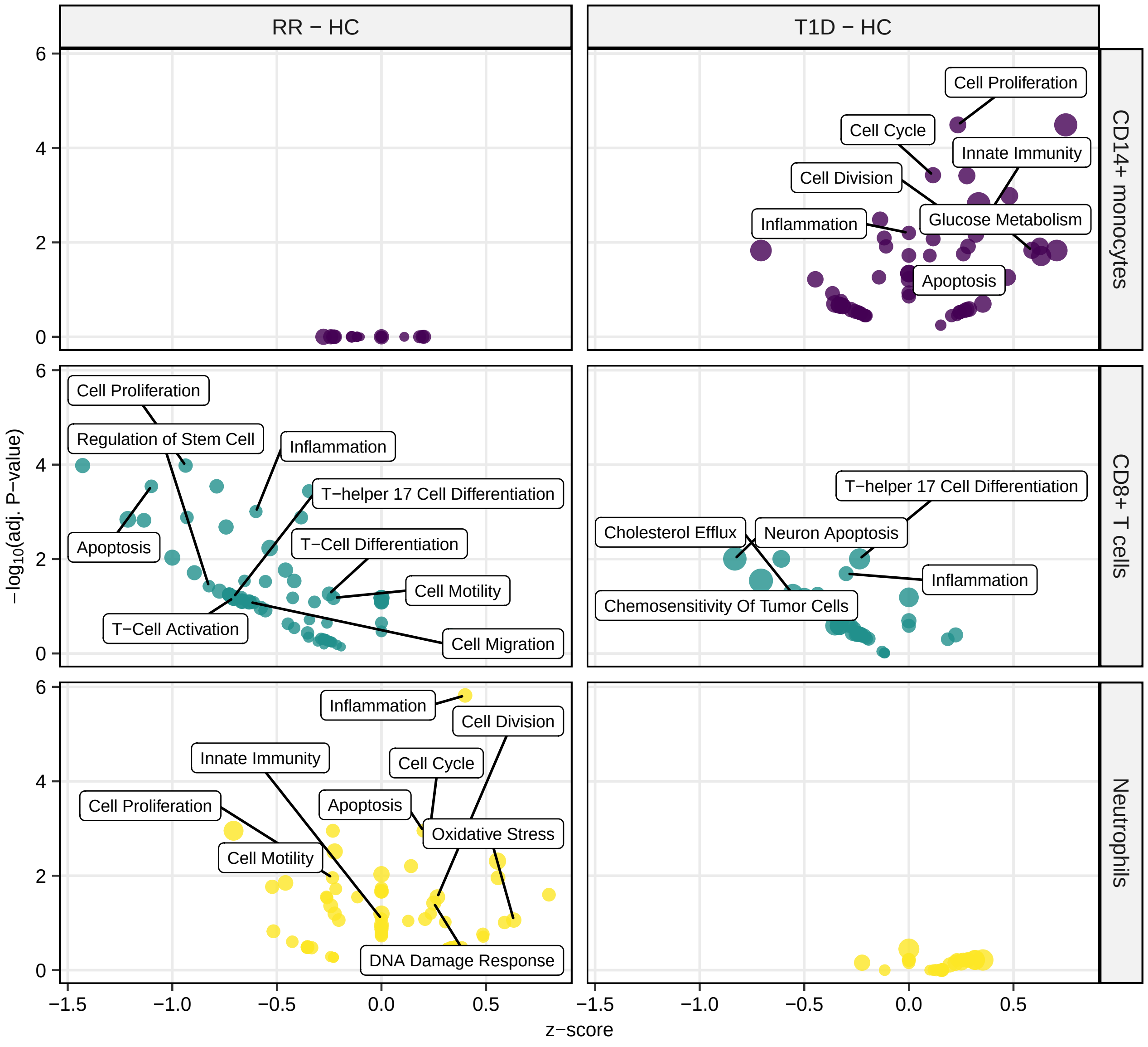

Figure S3: MiRNA functional enrichment analysis in MS and T1D. The panel shows the enriched categories resulting from the over-representation analysis of differentially expressed miRNAs in each cell type and for each comparison. The y-axis represents the negative logarithm of the Benjamini-Hochberg adjusted p-value, while the x-axis shows the z-score of the enrichment, defined as $\frac{u-d}{\sqrt{n}}$, where u is the number of upregulated miRNAs belonging to the category, d is the number of downregulated miRNAs belonging to the category, and n is the total size of the category.

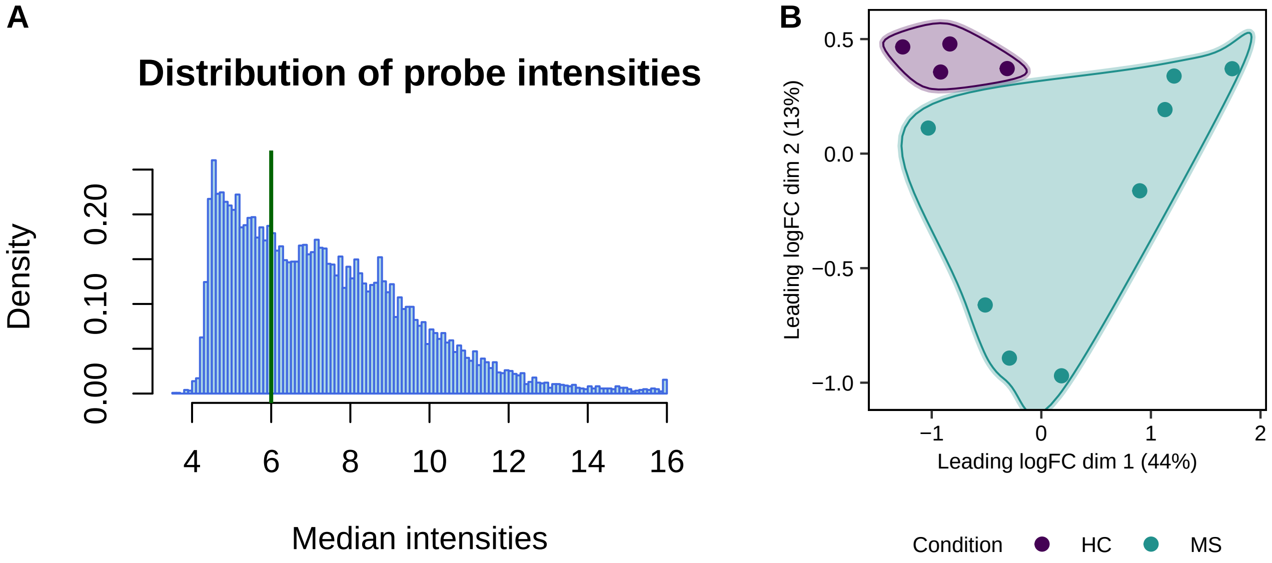

Figure S4: Pre-processing of CD8$^+$ transcriptomes. **A** shows the density of median probe intensities for each gene. To remove the majority of lowly expressed genes, we used a cutoff value of 6. **B** illustrates that gene expression of CD8$^+$ T cells in the MDS space is mainly affected by disease state.

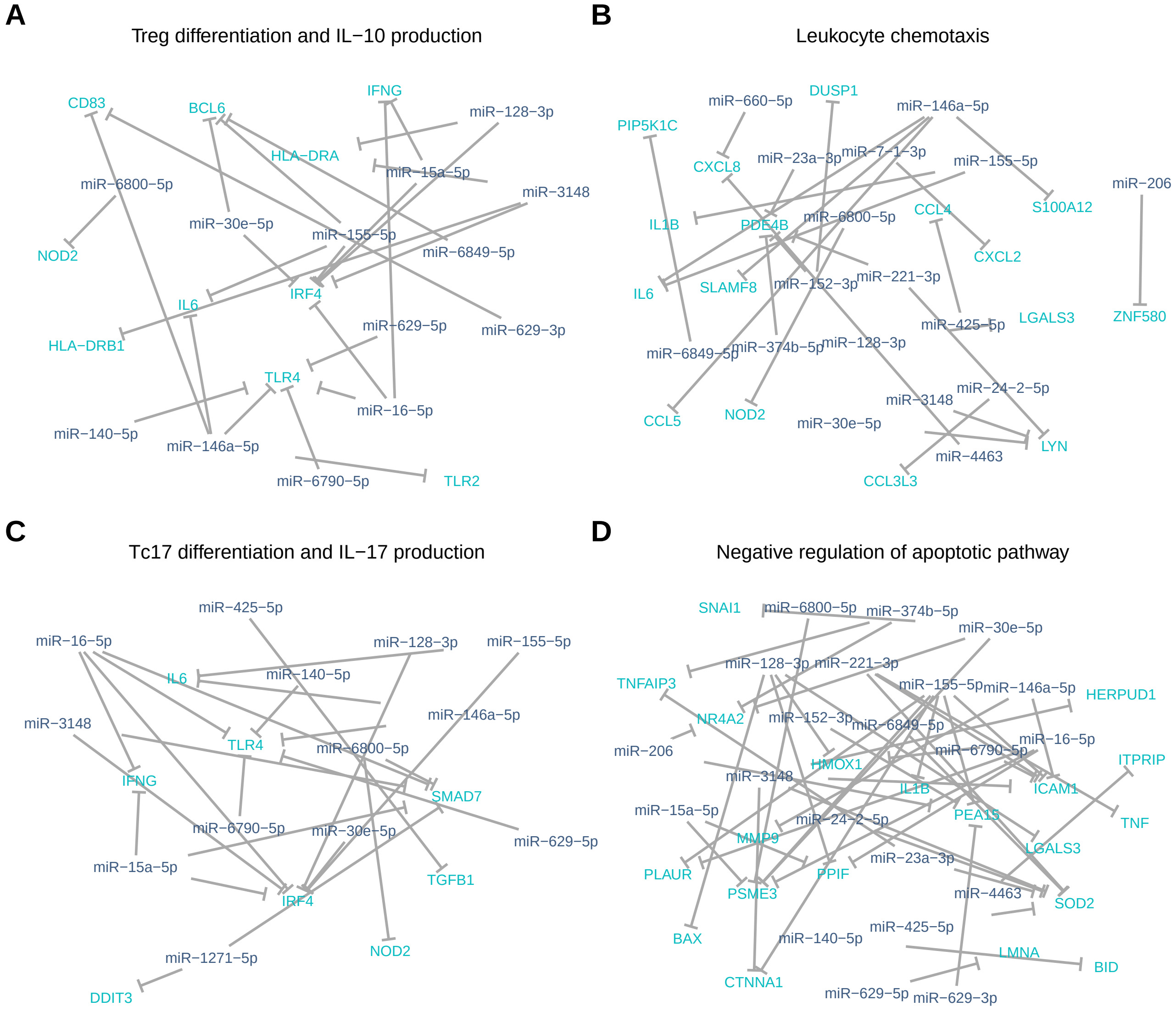

Figure S5: Influential miRNA–target interactions in CD8^{+} T cells from RRMS patients. **A** shows influential pairs associated with Treg differentiation and IL-10 production. **B** shows influential pairs linked to leukocyte chemotaxis. **C** shows influential pairs implicated in Tc17 differentiation and IL-17 secretion. **D** shows influential pairs involved in the negative regulation of apoptotic pathways.

A

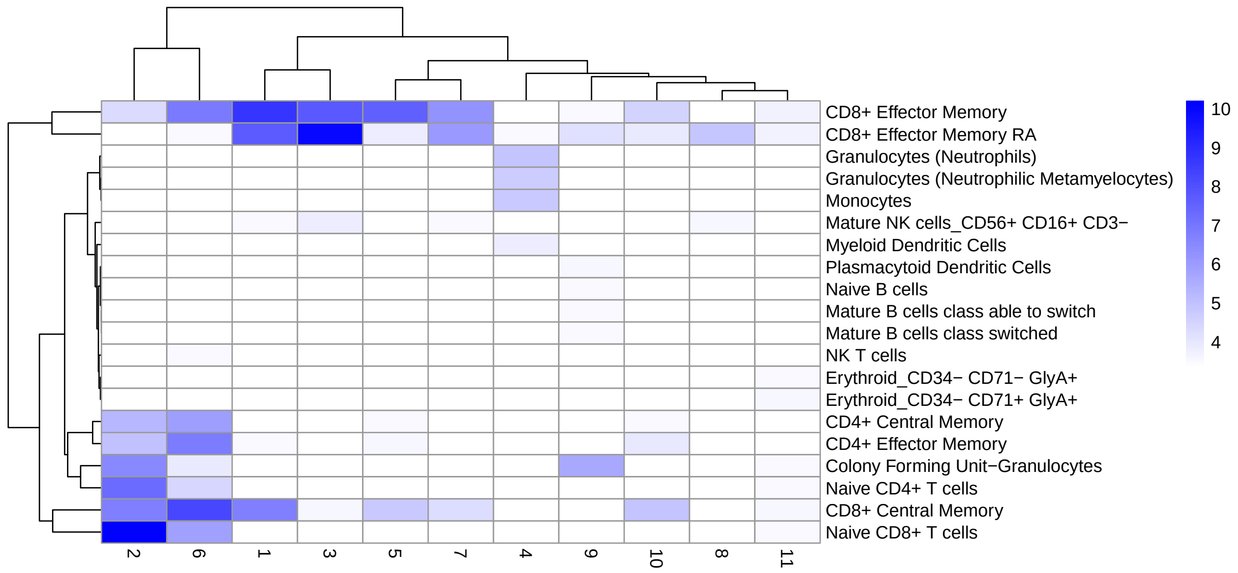

B

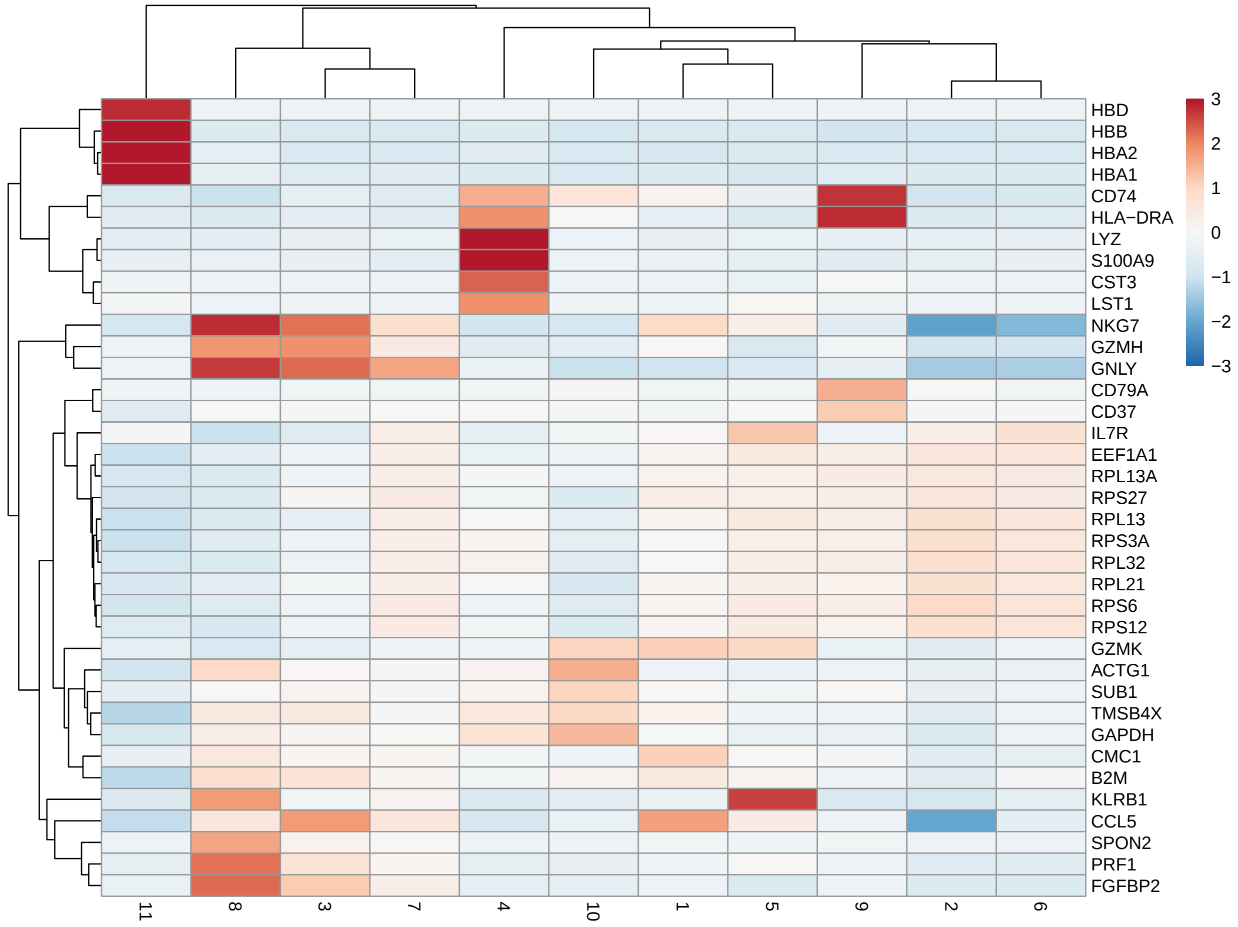

Figure S6: Cluster annotation of single-cell CD8$^+$ transcriptomes. **A** displays the score of cluster identity predictions according to the SingleR method using the Novershtern reference. **B** shows the expression of the top marker genes for each cluster.

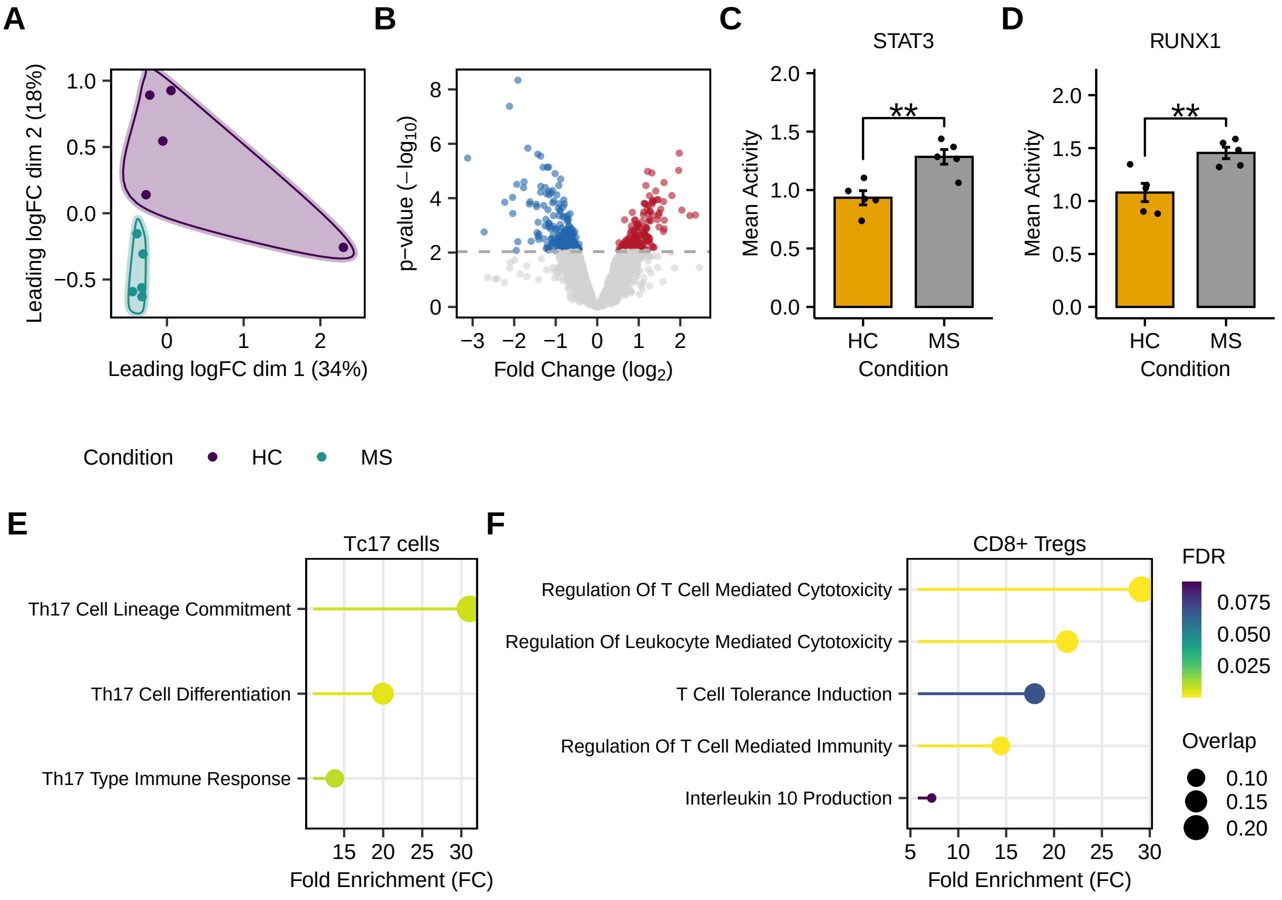

Figure S7: Pseudobulk analysis and identification of Tc17 cells and CD8$^+$ Tregs. **A** shows the variability of gene expression in pseudobulks of CD8$^+$ T cells. **B** represents DEGs derived from differential expression analysis of pseudobulks between MS patients and controls. **C** and **D** show the overactivation of STAT3 and RUNX1, respectively, based on estimated transcriptional activities. **E** shows the enrichment of IL-17 related categories in the identified Tc17 population. **F** shows the enrichment of regulatory categories in the identified CD8$^+$ Treg subset.

Cell Type	HSG_HC	HSG_RR	HSR_HC	HSR_T1D
$CD14^+$ monocytes	2	2	1	1
$CD8^+$ cells	2	2	1	0
neutrophils	3	1	1	1

Table S1: Excluded samples for each cell type and condition.

Table S2–S10 (Online Material)

The online supplementary material includes Tables S2–S10, which provide extended quantitative results not shown in the main manuscript. Specifically:

- **Tables S2–S4** contain the lists of differentially expressed miRNAs identified in $CD8^+$ T cells, $CD14^+$ monocytes, and neutrophils from MS patients.
- **Tables S5–S7** contain the lists of differentially expressed miRNAs identified in $CD8^+$ T cells, $CD14^+$ monocytes, and neutrophils from T1D patients.
- **Table S8** lists the DEGs identified in $CD8^+$ T cells from MS patients.
- **Table S9** lists the DEGs identified through pseudobulk differential expression analysis of $CD8^+$ T cells from MS patients.
- **Table S10** contains the primer sequences used for the RT-qPCR experiments presented in the manuscript.
